# Differential integrated stress response and asparagine production drive symbiosis and therapy resistance of pancreatic adenocarcinoma cells

**DOI:** 10.1038/s43018-022-00463-1

**Published:** 2022-11-21

**Authors:** Christopher J. Halbrook, Galloway Thurston, Seth Boyer, Cecily Anaraki, Jennifer A. Jiménez, Amy McCarthy, Nina G. Steele, Samuel A. Kerk, Hanna S. Hong, Lin Lin, Fiona V. Law, Catherine Felton, Lorenzo Scipioni, Peter Sajjakulnukit, Anthony Andren, Alica K. Beutel, Rima Singh, Barbara S. Nelson, Fran Van Den Bergh, Abigail S. Krall, Peter J. Mullen, Li Zhang, Sandeep Batra, Jennifer P. Morton, Ben Z. Stanger, Heather R. Christofk, Michelle A. Digman, Daniel A. Beard, Andrea Viale, Ji Zhang, Howard C. Crawford, Marina Pasca di Magliano, Claus Jorgensen, Costas A. Lyssiotis

**Affiliations:** 1 Department of Molecular Biology and Biochemistry, University of California Irvine, Irvine, CA USA; 2 University of California Irvine Chao Family Comprehensive Cancer Center, Orange, CA USA; 3 Department of Molecular & Integrative Physiology, University of Michigan, Ann Arbor, MI USA; 4 Cancer Research UK Manchester Institute, University of Manchester, Manchester, UK; 5 Department of Surgery, University of Michigan, Ann Arbor, MI USA; 6 Department of Biomedical Engineering, University of California Irvine, Irvine, CA USA; 7 Department of Biological Chemistry, University of California Los Angeles, Los Angeles, CA USA; 8 Riley Hospital for Children at Indiana University Health, Indianapolis, IN USA; 9 Cancer Research UK Beatson Institute and Institute of Cancer Sciences, University of Glasgow, Glasgow, UK; 10 Gastroenterology Division, Department of Medicine, University of Pennsylvania, Philadelphia, PA USA; 11 Department of Genomic Medicine, Division of Cancer Medicine, The University of Texas MD Anderson Cancer Center, Houston, TX USA; 12 Herman B. Wells Center for Pediatric Research, Indiana University School of Medicine, Indianapolis, IN USA; 13 University of Michigan Rogel Cancer Center, University of Michigan, Ann Arbor, MI USA; 14 Department of Internal Medicine, Division of Gastroenterology and Hepatology, University of Michigan, Ann Arbor, MI USA; 15 Present Address: Department of Molecular Biology and Biochemistry, University of California Irvine, Irvine, CA USA; 16 Present Address: Department of Surgery, Henry Ford Health System, Detroit, MI USA

**Keywords:** Tumour heterogeneity, Cancer metabolism, Pancreatic cancer, Cancer

## Abstract

The pancreatic tumor microenvironment drives deregulated nutrient availability. Accordingly, pancreatic cancer cells require metabolic adaptations to survive and proliferate. Pancreatic cancer subtypes have been characterized by transcriptional and functional differences, with subtypes reported to exist within the same tumor. However, it remains unclear if this diversity extends to metabolic programming. Here, using metabolomic profiling and functional interrogation of metabolic dependencies, we identify two distinct metabolic subclasses among neoplastic populations within individual human and mouse tumors. Furthermore, these populations are poised for metabolic cross-talk, and in examining this, we find an unexpected role for asparagine supporting proliferation during limited respiration. Constitutive GCN2 activation permits ATF4 signaling in one subtype, driving excess asparagine production. Asparagine release provides resistance during impaired respiration, enabling symbiosis. Functionally, availability of exogenous asparagine during limited respiration indirectly supports maintenance of aspartate pools, a rate-limiting biosynthetic precursor. Conversely, depletion of extracellular asparagine with PEG–asparaginase sensitizes tumors to mitochondrial targeting with phenformin.

## Abstract (web summary)

Halbrook et al. report two metabolic subgroups of pancreatic ductal adenocarcinoma cells defined by differential integrated stress response and asparagine production that permit symbiosis and phenformin resistance.

## Main

Pancreatic ductal adenocarcinoma (PDA) remains one of the deadliest major cancers, contrasting a relatively low incidence rate^1^. The primary reasons for this are related to the difficulty with early detection and a lack of effective therapeutic options. A principal barrier to treatment of pancreatic cancer is the densely fibrotic tumor microenvironment, the high interstitial pressure of which acts to collapse blood vessels and impair the delivery of chemotherapy^2^. This lack of functional vasculature leads to deregulated nutrient availability within the tumor^3^, causing cancer cells to develop numerous metabolic adaptations to allow for proliferation under hypoxic and austere conditions^4^.

Most PDAs express mutant *KRAS*^5^, so early efforts to understand metabolism in pancreatic cancer focused on the cell-intrinsic metabolic rewiring downstream of KRAS signaling. Modulating oncogenic *KRAS* expression in pancreatic cancer cells demonstrated distinct rewiring of central carbon metabolism to shift glycolytic intermediates into bioenergetic pathways and a preferential use of glutamine for tricarboxylic acid (TCA) cycle metabolism^6,7^. In addition, metabolic profiling of a panel of PDA cell lines revealed heterogeneity among preferred bioenergetic pathways and sensitivity to metabolic inhibitors^8^. However, the malignant cancer cells themselves reside within a tumor microenvironment that is a complex ecosystem^9^, with diverse populations of fibroblasts and immune cells functioning to create a niche supporting cancer cell survival and tumor growth^10^.

Accordingly, this allows for many and varied cooperative intratumoral cross-talk interactions^9,10^. These include the direct metabolic support of cancer cells from adjacent stromal and immune cells. For example, PDA cells have been shown to use fibroblast-derived alanine and lysophosphatidylcholines to serve as alternate carbon and lipid fuel sources^11,12^, respectively. These metabolic support mechanisms can also directly contribute to PDA therapeutic resistance. Tumor-associated macrophages and fibroblasts have been found to release pyrimidines, including deoxycytidine, providing chemoresistance through molecular competition of antimetabolite gemcitabine commonly used in PDA treatment^13,14^. PDA-derived metabolites also function as part of the molecular signaling used to polarize tumor-associated macrophages through lactate secretion^15^, further demonstrating the reciprocal nature of the metabolic symbiosis in the pancreatic tumor microenvironment.

Recently it has become apparent that even the previous recognition of heterogeneity of the pancreatic tumor microenvironment has been underappreciated. Numerous fibroblast populations have been described with distinct functions, including structural support and immune modulation and potential roles in antigen presentation^16–18^. Further, several studies have also demonstrated spatial heterogeneity in pancreatic tumors, with distinct immune and stromal behaviors present in different regions of individual tumors^19,20^.

Further, the malignant populations within individual tumors are not homogenous^21–23^. In fact, different clonally isolated populations of PDA have been shown to engage lineage-specific behavior, such as the ability to coordinate a strongly immunosuppressive tumor microenvironment^24^. Accordingly, we postulated that epithelial heterogeneity might also result in differential metabolic programming across different cancer cell populations within individual tumors. Furthermore, given the diversity of the neoplastic cell populations found in pancreatic tumors, we postulated that clonal differences among cancer cells within the same tumor might also extend to metabolic behaviors capable of symbiotic support^25^.

## Results

### Characterization stratifies two metabolic classes of PDA

**Fig. 1:**
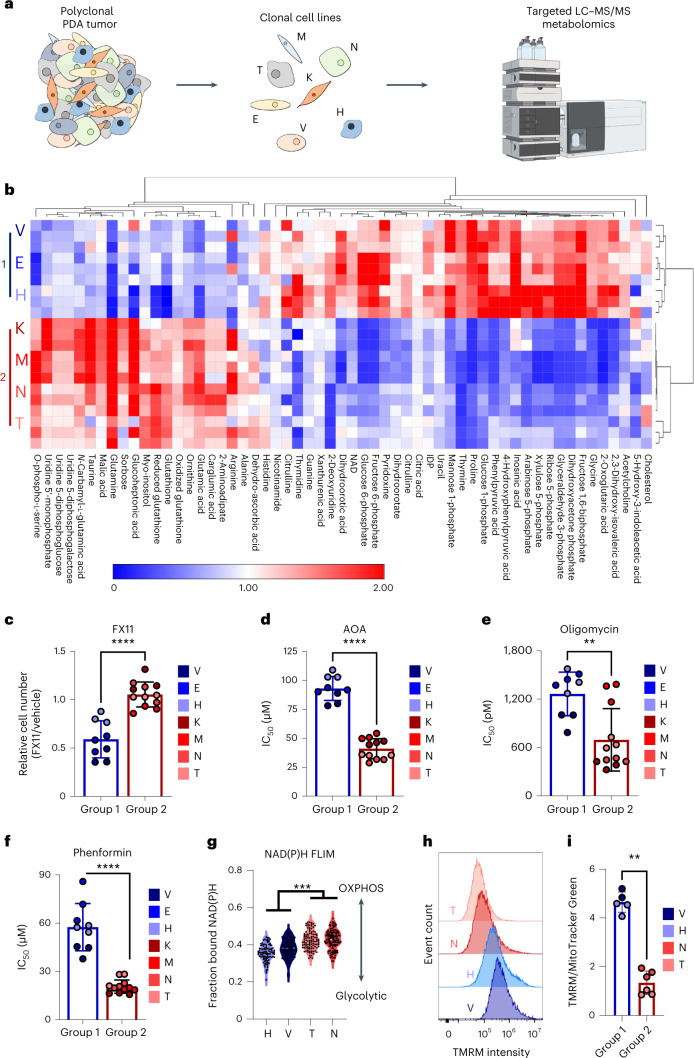
Metabolic characterization reveals two distinct cellular populations from PDA tumors. **a**, A polyclonal cell line established from a mouse pancreatic tumor was subcloned into seven clonal lines and subjected to LC–MS/MS metabolomics analysis. **b**, Heat map representation of significantly different intracellular metabolites pooled from a positive and negative ionization mode analysis among the clonal cell lines grown under the same media conditions; fold change of ±2; *P* = 0.01 between group 1 clones (E, V and H) and group 2 clones (K, M, N and T). Rows are clonal cell lines in triplicate, and columns represent metabolites (*n* = 3 biological replicates per cell line). **c**–**f**, Sensitivity of clonal cell lines to 33 µM FX11 (**c**) and IC_50_ values for aminooxyacetic acid (AOA) (**d**), oligomycin (**e**) and phenformin (**f**); *n* = 3 biological replicates per cell line. **g**, Group 1 clones (H and V) and group 2 clones (N and T) were subjected to NAD(P)H FLIM. Data are presented as a ratio of free to protein-bound NAD(P)H; *n* = 104 clone H cells, *n* = 108 clone V cells, *n* = 136 clone N cells and *n* = 105 clone T cells; OXPHOS, oxidative phosphorylation. **h**, Histograms of the indicated clones stained with TMRM and analyzed via flow cytometry. **i**, Ratio of TMRM to MitoTracker Green staining of the indicated clonal lines (*n* = 2 biological replicates for clone V and *n* = 3 biological replicates for clones H, N and T). Error bars represent mean ± s.d.; **P ≤ 0.01, ***P ≤ 0.001 and ****P ≤ 0.0001 by two-tailed Mann–Whitney test (**c**–**f** and **i**) or one-way ANOVA with a Tukey post hoc test; *P* < 0.0001 (**c**); *P* < 0.0001 (**d**); *P* = 0.0018 (**e**); *P* < 0.0001 (**f**); H versus T *P* < 0.0001, H versus N *P* < 0.0001, V versus T *P* < 0.0001 and V versus N *P* = 0.0003 (**g**); *P* = 0.0043 (**i**). Source data

**Extended Data Fig. 1:**
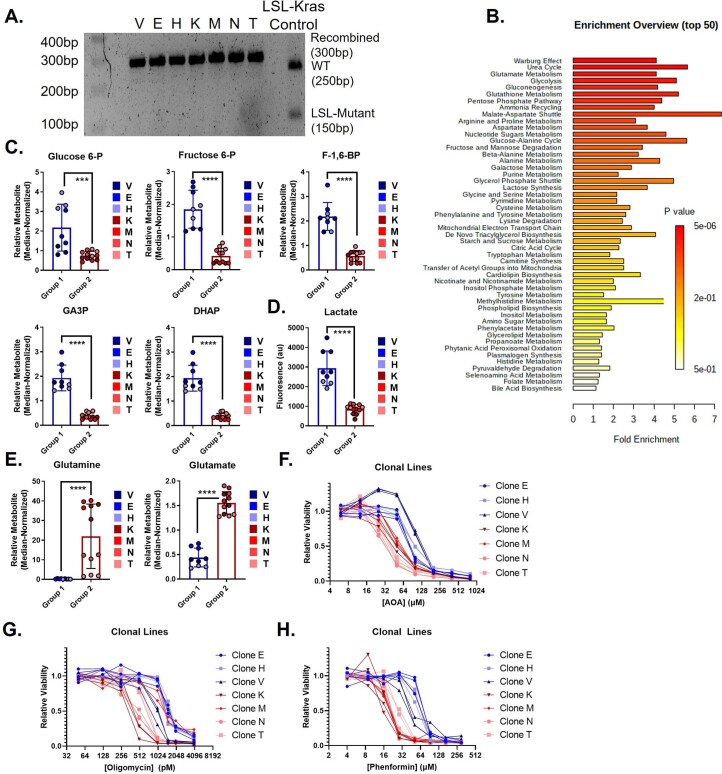
Enriched Metabolic Pathways Present in Clonal Populations. **A**. Agarose gel visualization of PCR products for recombination of Kras^G12D^ allele in clonal lines vs. Kras^LSL-G12D/+^ mouse tail control DNA. **B**. Top 50 pathways identified by MetaboAnalyst among metabolites differentially represented between group 1 and group 2 clones. **C**. Relative abundance of metabolites from glycolysis between clonal populations (n = 3 replicates per cell line). F-1,6-BP = fructose 1,6-bisphsophate, GA3P = glyceraldehyde-3-phosphate, DHAP = dihydroxyacetone phosphate. **D**. Lactate production of clonal cell lines (n = 3 replicates per cell line). **E**. Relative abundance of glutamine and glutamate between clonal populations (n = 3 replicates per cell line). **F**. Amniooxyacetic acid (AOA) dose response curves used to generate IC_50_ values in Fig. 1D. **G**. Oligomycin dose response curves used to generate IC_50_ values in Fig. 1E. **H**. Phenformin dose response curves used to generate IC_50_ values in Fig. 1F. Each line represents an independent dose response analysis. Error bars are mean ± SD, *** *P* ≤ 0.001; **** *P* ≤ 0.0001 by two-tailed Mann–Whitney test. Source data

To identify clonal differences in metabolism, we profiled a series of clonal cell lines derived from a single *Kras*^+/G12D^; *Trp53*^+/R172H^; *Pdx1*-Cre (KPC) mouse tumor (Fig. 1a and Supplemental Table 1) by liquid chromatography–tandem mass spectrometry (LC–MS/MS)-based metabolomics. The LSL-*Kras*^G12D^ allele was recombined in all the clones, demonstrating that they were derived from epithelial lineages (Extended Data Fig. 1a). Through unbiased clustering of the metabolomics analysis, we observed that the clones clearly separated into two distinct groups based on their steady-state metabolite pools (Fig. 1b). Pathway enrichment analysis demonstrated that these differences extended across several pathways, with glycolysis and Warburg metabolism topping the list (Extended Data Fig. 1b). Indeed, we found that clones V, E and H (hereafter termed group 1) demonstrated increased levels of glycolytic metabolites (Extended Data Fig. 1c) compared to clones K, M, N and T (termed group 2).

We also found enrichment of glycolytic intermediates correlated with enhanced lactate production in the conditioned medium of group 1 clones (Extended Data Fig. 1d). By contrast, we observed that group 2 clones had elevated pools of glutamine and glutamate relative to group 1 clones (Extended Data Fig. 1e). This observation was intriguing, as we have previously found that PDA cells in vitro use glutamine-derived carbon to fuel TCA cycle metabolism^6,7^. Given the differences in the glycolytic versus putative mitochondrial metabolites, we speculated that the clonal populations might exhibit differential dependency on these pathways.

Accordingly, we next investigated if the differences in baseline metabolism correlated with metabolic vulnerabilities between these groups. Indeed, we observed a difference in sensitivity to inhibition of glycolysis in group 1 clones following treatment with the lactate dehydrogenase inhibitor FX11 (Fig. 1c), and group 2 clones were more sensitive to transaminase inhibition (Fig. 1d and Extended Data Fig. 1f). Accordingly, as we hypothesized that glutamine was used to fuel mitochondrial metabolism, we tested if this differential sensitivity would extend to direct inhibition of mitochondrial respiration. Indeed, we observed that group 2 clones were more sensitive to inhibition of mitochondrial respiration through treatment with either the ATP synthase inhibitor oligomycin or the complex I inhibitor phenformin (Fig. 1e,f and Extended Data Fig. 1g,h).

**Extended Data Fig. 2:**
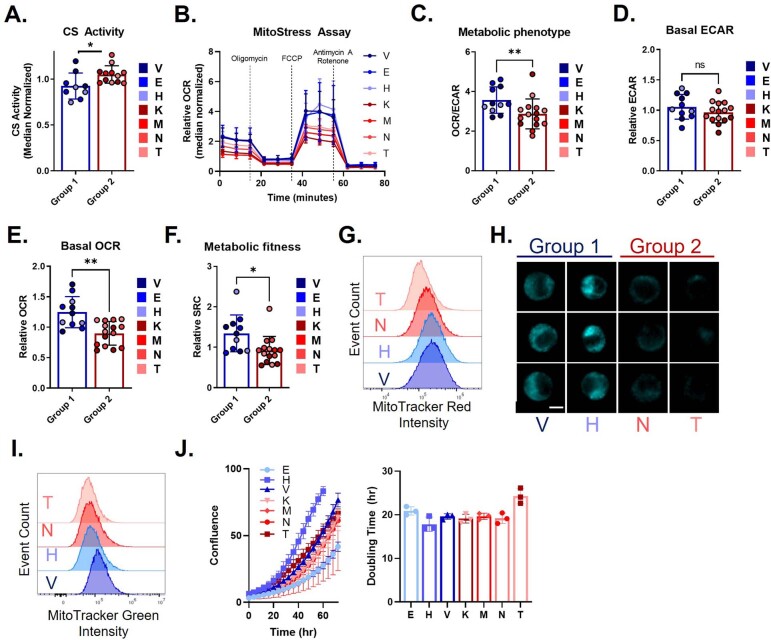
Clonal Metabolic Phenotypes. **A**. Citrate Synthase (CS) activity of clonal lines (n = 3 per clone). **B**. MitoStress profile of the oxygen consumption rate (OCR) over time, treated with (2 μM), FCCP (0.5-2 μM), and rotenone/antimycin A (1 μM) at the indicated time points. Data are average of technical replicates normalized to independent experiments for each clone (n = 4 clone V, n = 4 clone E, n = 3 clone H, n = 5 clone N, n = 4 clone K, n = 2 clone M, n = 4 clone T). **C**. Metabolic phenotypes of clonal cell lines defined as the ratio of the OCR to extracellular acidification rate (ECAR) (n = 4 clone V, n = 4 clone E, n = 3 clone H, n = 4 clone K, n = 2 clone M, n = 5 clone N, n = 4 clone T). **D**. Basal ECAR of clonal lines (n = 4 clone V, n = 4 clone E, n = 3 clone H, n = 4 clone K, n = 2 clone M, n = 5 clone N, n = 4 clone T). **E**. Basal OCR of clonal lines (n = 4 clone V, n = 4 clone E, n = 3 clone H, n = 4 clone K, n = 2 clone M, n = 5 clone N, n = 4 clone T). **F**. Metabolic fitness as defined by the spare respiratory capacity of clonal cell lines (n = 4 clone V, n = 4 clone E, n = 3 clone H, n = 4 clone K, n = 2 clone M, n = 5 clone N, n = 4 clone T). **G**. Histogram representation of the MitoTracker Red intensity of group 1 clones (H,V) and group 2 clones (T,N). **H**. Representative images of MitoTracker Red staining at the mean fluorescence index (MFI) of **I**. Scale bar = 7 µm. **J**. Histogram representation of the MitoTracker Green intensity of group 1 clones (H,V) and group 2 clones (T,N). **K**. Doubling times of the individual clone lines (n = 3 per clone). Error bars are mean ± SD, * *P* ≤ 0.05; ** *P* ≤ 0.01; *** *P* ≤ 0.001; **** *P* ≤ 0.0001 by two-tailed Mann–Whitney test. Source data

To examine the bioenergetic state of the clonal cell lines under normal growth conditions, we used a phasor fluorescence lifetime imaging (FLIM) assay of NADH. The fluorescence lifetime of NADH is longer when bound to enzymes involved in mitochondrial metabolism (~3.4 ns) in contrast to free cytoplasmic NAPH (~0.4 ns)^26^, and the ratio of these values correlates to glycolytic versus mitochondrial metabolism in cells. As predicted, we found that a set of group 1 clones have less protein-bound NADH, indicative of a more glycolytic phenotype, while a set of group 2 clones have higher levels of protein-bound NADH, demonstrating a preference for mitochondrial metabolism (Fig. 1g). Consistently, we observed a modest, but statistically significantly, increase in citrate synthase activity (a readout of mitochondrial metabolism) in group 2 clones compared to group 1 clones (Extended Data Fig. 2a). Unexpectedly, we did observe an increased metabolic phenotype in the group 1 clones as measured by a Seahorse instrument (Extended Data Fig. 2b,c), as the higher trend in extracellular acidification rate in the group 1 clones was accompanied by an increased oxygen consumption rate (Extended Data Fig. 2d,e). We also observed an increase in the spare respiratory capacity in the group 1 clones compared to in the group 2 clones (Extended Data Fig. 2f), potentially suggesting that the group 1 clones are more bioenergetic in general.

To better understand this increase in spare respiratory capacity, we measured mitochondrial potential across a representative set of clones. Both tetramethylrhodamine methyl ester perchlorate (TMRM) and MitoTracker Red staining revealed that group 1 clones have higher mitochondrial potential (Fig. 1h and Extended Data Fig. 2g,h). Importantly, the higher intensity of TMRM staining in group 1 clones was maintained when normalized for total mitochondrial mass (Fig. 1i and Extended Data Fig. 2i). Taken together, the higher spare respiratory capacity and elevated mitochondrial potential may in part explain the lower sensitivity of the group 1 clones to mitochondrial inhibition than that observed in group 2 clones. Importantly, while we noted small differences in the growth rates of the clonal cell lines, these did not correlate with the identified clusters (Extended Data Fig. 2j).

### Clonal cross-talk provides metabolic support

**Fig. 2:**
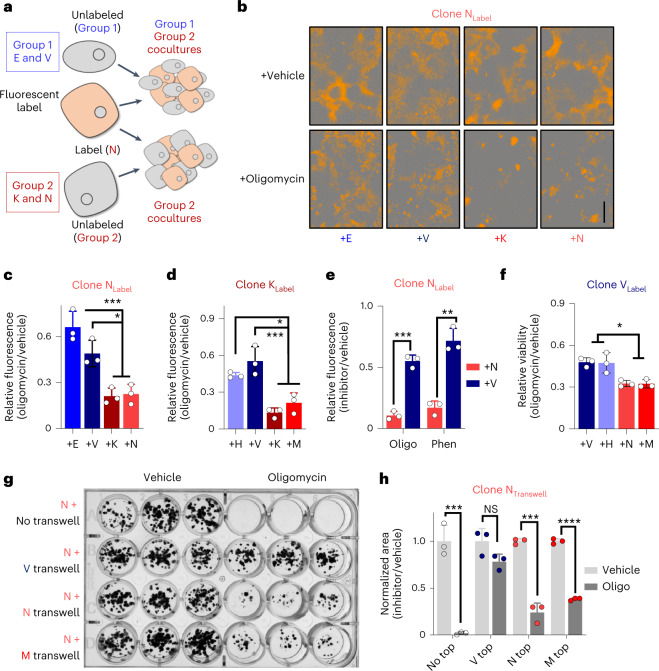
Cocultures reveal cross-talk interactions between clonal groups. **a**, Clonal line N was encoded with a fluorescent label, plated in direct coculture with unlabeled clones and treated with oligomycin or vehicle. **b**, Representative images (one of three biological replicates) of a labeled oligomycin-sensitive clone N cocultured with unlabeled resistant clones (E and V) or with unlabeled sensitive clones (K and N) and treated with oligomycin or vehicle; scale bar, 400 µm. **c**, Fluorescent plate area of oligomycin-treated (0.75 nM) versus vehicle-treated N-labeled cocultures (*n* = 3 samples). **d**, Fluorescent plate area of oligomycin-treated (1 nM) versus vehicle-treated K-labeled cocultures with unlabeled resistant clones (H and V) or with unlabeled sensitive clones (K and M; *n* = 3 samples). **e**, Fluorescent plate area of 1 nM oligomycin (Oligo)- or 25 µM phenformin (Phen)-treated versus vehicle-treated cocultures for labeled sensitive clone N cocultured with unlabeled clone N or insensitive clone V (*n* = 3 samples). **f**, Fluorescent plate area of the 1.5 nM oligomycin-treated cocultures relative to vehicle-treated cocultures (*n* = 3 samples). **g**, Group 2 clone N was plated with no transwell or with transwells containing group 1 clone V or group 2 clones N and M. Cultures were treated with 0.25 nM oligomycin with fresh drug added every 48 h for 10 d and were fixed and stained with crystal violet. The image is representative of three experimental repeats. **h**, Quantitation of colony area of **g** (*n* = 3 samples). Error bars represent mean ± s.d.; * *P* ≤ 0.05, ** *P* ≤ 0.01, *** *P* ≤ 0.001 and **** *P* ≤ 0.0001 by one-way ANOVA with a Tukey post hoc test (**c**, **d** and **f**) or by two-tailed Student’s *t* test (**e** and **h**); NS, not significant; +E versus +K *P* = 0.0007, +E versus +N *P* = 0.0009, +V versus +K *P* = 0.0157 and +V versus +N *P* = 0.0207 (**c**); +H versus +K *P* = 0.0047, +H versus +M *P* = 0.0266, +V versus +K *P* = 0.0006 and +V versus +M *P* = 0.0024 (**d**); oligomycin *P* = 0.0002 and phenformin *P* = 0.0012 (**e**); +V versus +N *P* = 0.0114, +V versus +M *P* = 0.0108, +H versus +N *P* = 0.0137 and +H versus +M *P* = 0.013 (**f**); no top *P* = 0.000585, V top *P* = 0.076023, N top *P* = 0.0002277 and M top *P* = 0.000004 (**g**). Source data

**Extended Data Fig. 3:**
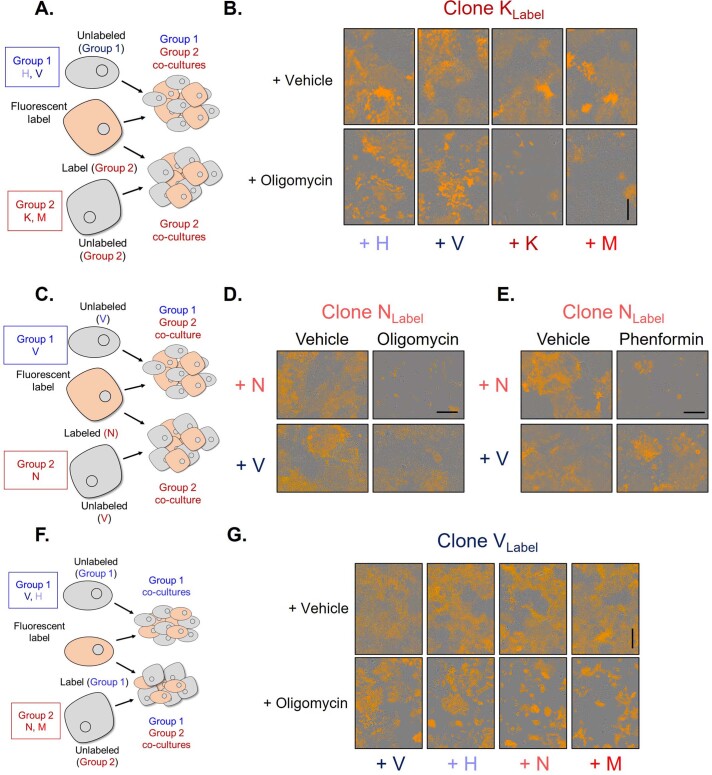
Co-Culture Metabolic Assays. **A**. Fluorescently labeled clone K co-cultures were treated with oligomycin or vehicle. **B**. Representative images from Fig. 2D of labeled K co-cultured with unlabeled resistant clones (H,V) or with unlabeled sensitive clones (K,M). **C**. Fluorescently labeled clone N co-cultures were treated with oligomycin, phenformin or vehicle. **D,E**. Representative images of 1 nM oligomycin or vehicle treated co-cultures with unlabeled resistant V (**D**), or unlabeled sensitive clones N (**E**) to generate data presented in Fig. 2E. **F**. Fluorescently labeled clone V co-cultures were treated with 1.5 nm oligomycin or vehicle. **G**. Representative images from Fig. 2F of labeled V co-cultured with unlabeled resistant clones (V,H) or with unlabeled sensitive clones (N,M). Error bars are mean ± SD, Scale Bars = 400 µm, * *P* ≤ 0.05 by one-way Anova with Tukey post hoc.

**Extended Data Fig. 4:**
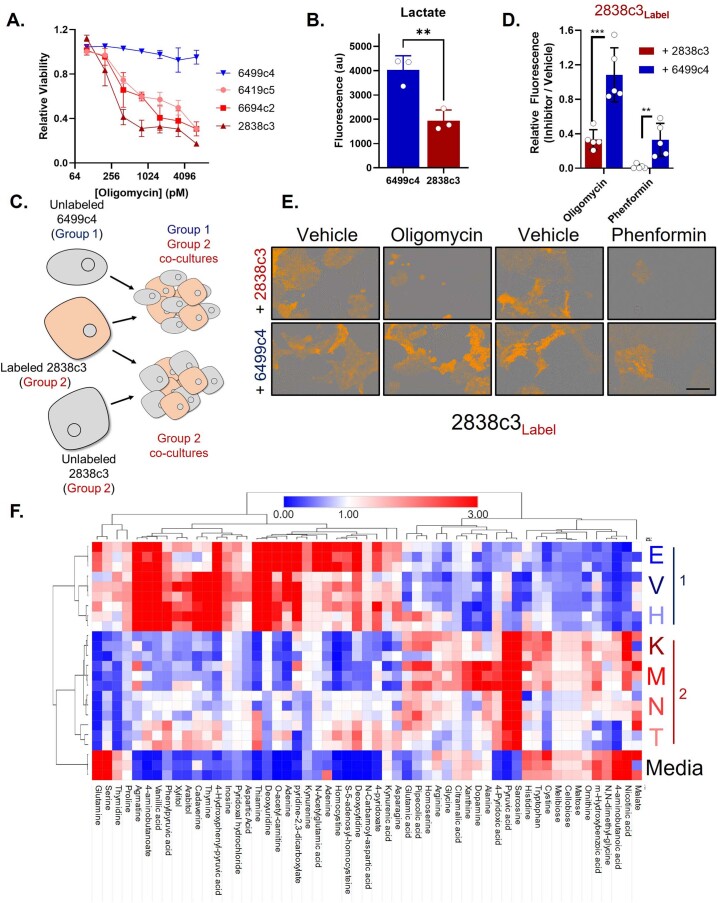
Consistent Metabolic Features in a Second PDA Clonal Cell Model and Media Metabolite Profiling. **A**. Dose response curves of oligomycin treated KPC clones 2838C3, 6499c4, 6419c5, and 6694c2 (n = 3 per clone). **B**. Lactate production by clones 2838c3 and 6499c4 (n = 3 per clone). **C**. Group 2 clone 2838c3 was labeled with a fluorescent probe and co-cultured with either unlabeled 2838c3 or with unlabeled group 1 clone 6499c4 and then treated with either phenformin, oligomycin, or vehicle. **D**. Viability of 5 nM oligomycin or 100 µM phenformin treated co-cultures relative vehicle (n = 5). **E**. Representative images from Extended Data Fig. 4D. **F**. Heatmap of unsupervised 2-dimensional hierarchical clustering for metabolites from conditioned media after 48 hours of culture with ±2 fold difference and p = 0.01 between groups (n = 3 per clone). Error bars are mean ± SD, Scale Bars = 400 µm, ** *P* ≤ 0.01 by two-tailed student’s t test. Source data

Next, we sought to determine if sensitive clones were protected from metabolic inhibition via metabolic support from less sensitive clones. To accomplish this, we fluorescently labeled a clonal line from one group and then cocultured it with either an unlabeled version of itself, a different clonal line from the same group or a clonal line from the opposite group (Fig. 2a). Using this strategy at a concentration of oligomycin sufficient to block the proliferation of group 2 cells, we demonstrated that the group 1 clones support the growth of oligomycin-treated group 2 clones (Fig. 2b,c). This was reproducible across clones (Fig. 2d and Extended Data Fig. 3a–d) and extended to the complex I inhibitor phenformin (Fig. 2e and Extended Data Fig. 3e). By contrast, proliferation of an oligomycin-treated group 1 clone was not similarly enhanced in coculture with a different group 1 clone, and oligomycin-treated group 1 clones were less proliferative when cocultured with group 2 clones (Fig. 2f and Extended Data Fig. 3f,g). To determine if our observations extended to other clonal PDA cell line models, we obtained a second set of mouse KPC clones^24^. Here again we observed differences in oligomycin sensitivity, differential lactate production and the rescue of mitochondrial inhibition in cocultures (Extended Data Fig. 4a–e).

### Asparagine supports proliferation during limited respiration

**Fig. 3:**
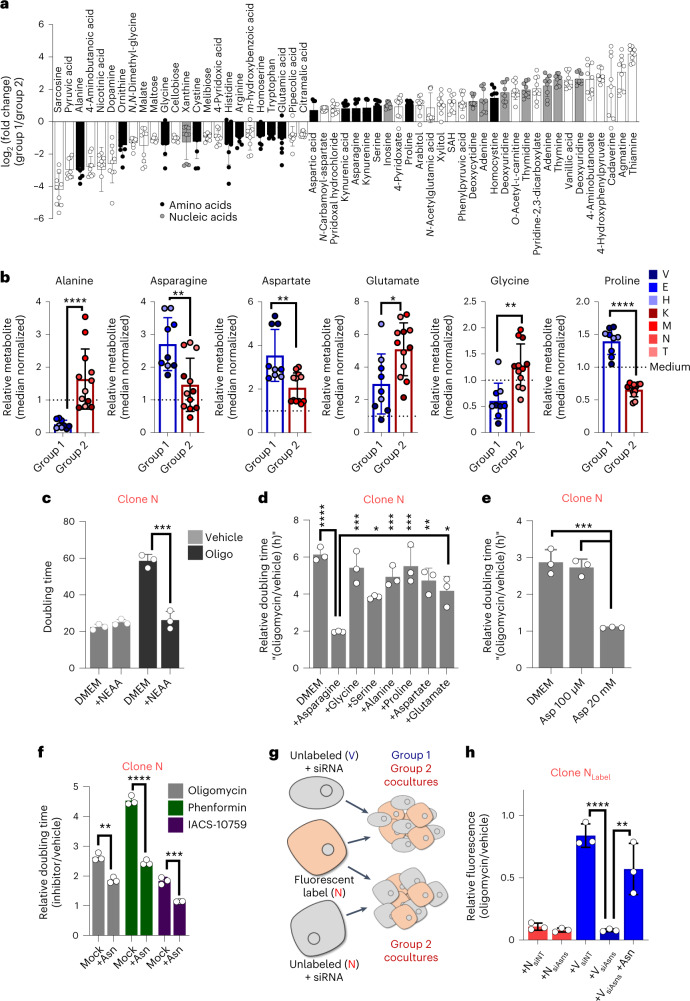
Media profiling reveals that asparagine rescues inhibition of respiration. **a**, Differentially consumed/released metabolites present in the media from group 1 and group 2 clones (*n* = 3 replicates per cell line) after 48 h of culture. **b**, Relative abundance of NEAAs present in conditioned medium at higher levels than in basal medium (*n* = 3 replicates per cell line). **c**, Doubling time of clone N treated with 1 nM oligomycin in the presence or absence of medium containing a 100 µM cocktail of all NEAAs (*n* = 3 samples per condition). **d**, Doubling times of clone N treated with 1 nM oligomycin in the presence or absence of individual NEAAs (*n* = 3 samples per condition). **e**, Doubling times of clone N treated with 1 nM oligomycin in the presence or absence of 100 µM or 20 mM aspartate (Asp; *n* = 3 samples per condition). **f**, Doubling times of clonal line N treated with 1 nM oligomycin, 25 µM phenformin or 25 nM IACS-10759 ± asparagine (Asn). **g**, Clonal line N was encoded with a fluorescent label and plated in direct coculture with unlabeled clones transfected with siRNA targeting *Asns* or non-targeted (NT) control. **h**, Fluorescent plate area of 1 nM oligomycin-treated cocultures of labeled clone N with unlabeled clone N or insensitive clone V (*n* = 3 samples) transfected with the indicated siRNA with or without the addition of exogenous asparagine. Error bars represent mean ± s.d.; * *P* ≤ 0.05, ** *P* ≤ 0.01, *** *P* ≤ 0.001 and **** *P* ≤ 0.0001 by two-tailed Mann–Whitney test (**b**), two-tailed Student’s *t* test (**c** and **f**) or one-way ANOVA with a Tukey post hoc test (**d**, **e** and **h**); alanine *P* < 0.0001, asparagine *P* = 0.0043, aspartate *P* = 0.0043, glutamate *P* = 0.0184, glycine *P* = 0.0013 and proline *P* < 0.0001 (**b**); *P* = 0.0008 (**c**); DMEM versus +asparagine *P* < 0.0001, +asparagine versus +glycine *P* = 0.0001), +asparagine versus +serine *P* = 0.0419, +asparagine versus +alanine *P* = 0.0008, +asparagine versus +proline *P* = 0.0001, +asparagine versus +aspartate *P* = 0.0016 and +asparagine versus +glutamate *P* = 0.0122 (**d**); DMEM versus aspartate 20 mM *P* = 0.0002 and aspartate 100 µM versus aspartate 20 mM *P* = 0.0004 (**e**); mock versus +asparagine for oligomycin *P* = 0.0011, phenformin *P* < 0.0001 and IACS-10759 *P* = 0.0006 (**f**); V siNT versus V siAsns *P* < 0.0001, V siAsns versus V siAsns + asparagine *P* = 0.0572 (**h**). Source data

Next, we used transwell coculture assays with which we demonstrated that the ability of group 1 cells to facilitate proliferation of oligomycin-sensitive group 2 cells was not contact dependent (Fig. 2g,h). Based on this, we hypothesized that clonal cross-talk was mediated through metabolite exchange between the group 1 and group 2 clones. Thus, we profiled the medium from each of the clonal cell lines by LC–MS/MS-based metabolomics and compared it to the base medium. Unsupervised clustering segmented the seven clonal lines into the same two groups observed following analysis of the intracellular metabolomes (Extended Data Fig. 4f and Supplemental Table 2). Importantly, there was a clear difference in the levels of consumption and release of several metabolites between these groups, largely represented by amino acids and nucleosides, including the production of several non-essential amino acids (NEAAs; Fig. 3a,b).

**Extended Data Fig. 5:**
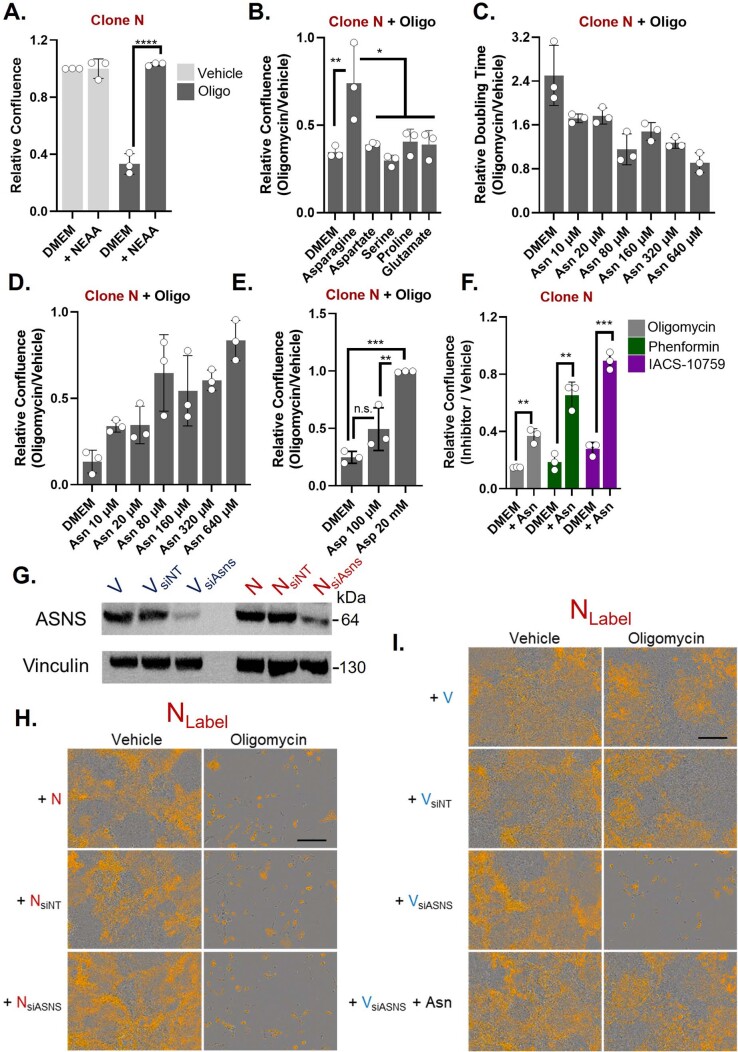
Asparagine Rescues the Inhibition of Respiration. **A**. Relative confluence of clone N treated with 1 nM oligomycin in the presence or absence of media containing a 100 µM cocktail of all NEAAs after 5 days (n = 3). **B**. Relative confluence of clone N treated with 1 nM oligomycin in the presence or absence of individual NEAAs after 5 days (n = 3). **C,D**. Dose dependent rescue of doubling time (**C**) and confluence after 5 days of culture (**D**) by exogenous asparagine (Asn) following treatment with 1 nM oligomycin. **E**. Relative confluence of clone N treated with 1 nM oligomycin in the presence or absence of 100 µM or 20 mM aspartate (n = 3). **F**. Relative confluence of clonal line N treated with 1 nM oligomycin, 25 µM phenformin, or 25 nM IACS-10759 after 5 days + /- Asn (n = 3). **G**. Immunoblot analysis of siRNA targeting *Asns* or non-targeted (NT) control in clonal line N or V. **H,I**. Representative images of vehicle vs 1 nM oligomycin treated co-cultures of labeled clone N with unlabeled clone N (**H**) or insensitive clone V (**I**) transfected with the indicated siRNA with or without the addition of exogenous Asn. Error bars are mean ± SD, Scale Bars = 400 µm, * *P* ≤ 0.05; ** *P* ≤ 0.01; *** *P* ≤ 0.001; **** *P* ≤ 0.0001 by two-tailed student’s t test (**A,F**), or one-way Anova with Tukey post hoc (**B,E**). Source data

Given these observations, we hypothesized that one or a combination of these amino acids may function to support metabolism in the presence of an inhibitor of mitochondrial respiration. Indeed, we found that treatment with a cocktail of NEAAs at 100 µM was sufficient to promote growth in the presence of oligomycin (Fig. 3c and Extended Data Fig. 5a). While aspartate has been identified as a limiting metabolite after inhibition of mitochondrial metabolism, we found that among the NEAAs, only asparagine was sufficient to rescue growth in the presence of mitochondrial inhibition at this concentration^27^ (Fig. 3d and Extended Data Fig. 5b). Further, asparagine rescue of the oligomycin-mediated inhibition of proliferation was dose dependent (Extended Data Fig. 5c,d) and well within the range of the physiological level in both sera and pancreatic tumor interstitial fluid^28^. We also demonstrated that aspartate is indeed able to rescue oligomycin-treated PDA cells at concentrations previously reported (that is, 20 mM (ref. ^29^); Fig. 3e and Extended Data Fig. 5e); however, this was well outside the physiological range of both circulating or intratumoral aspartate^28^. Further, in addition to oligomycin, we found that asparagine was able to restore growth in the presence of phenformin and IACS-10759, a distinct complex I inhibitor (Fig. 3f and Extended Data Fig. 5f).

To demonstrate that asparagine mediates the rescue of proliferation of sensitive clones in cocultures, we targeted the gene encoding the asparagine biosynthetic enzyme asparagine synthetase (*Asns*) in group 1 cells using RNA-mediated interference (RNAi) and then used our labeled coculture model (Fig. 3g and Extended Data Fig. 5g). As expected, addition of group 2 cells did not rescue the proliferation of oligomycin-treated labeled group 2 cells, whereas group 1 clones transfected with the non-targeting short interfering RNA (siRNA) provided a robust rescue (Fig. 3h and Extended Data Fig. 5h,i). However, this rescue was completely abolished by silencing *Asns* in the group 1 clone. Lastly, direct add back of asparagine to the group 2–group 1/siAsns coculture rescued the proliferation, pinpointing asparagine as the relevant factor mediating this metabolic cross-talk.

### Group 1 clones show constitutive integrated stress response (ISR)

**Fig. 4:**
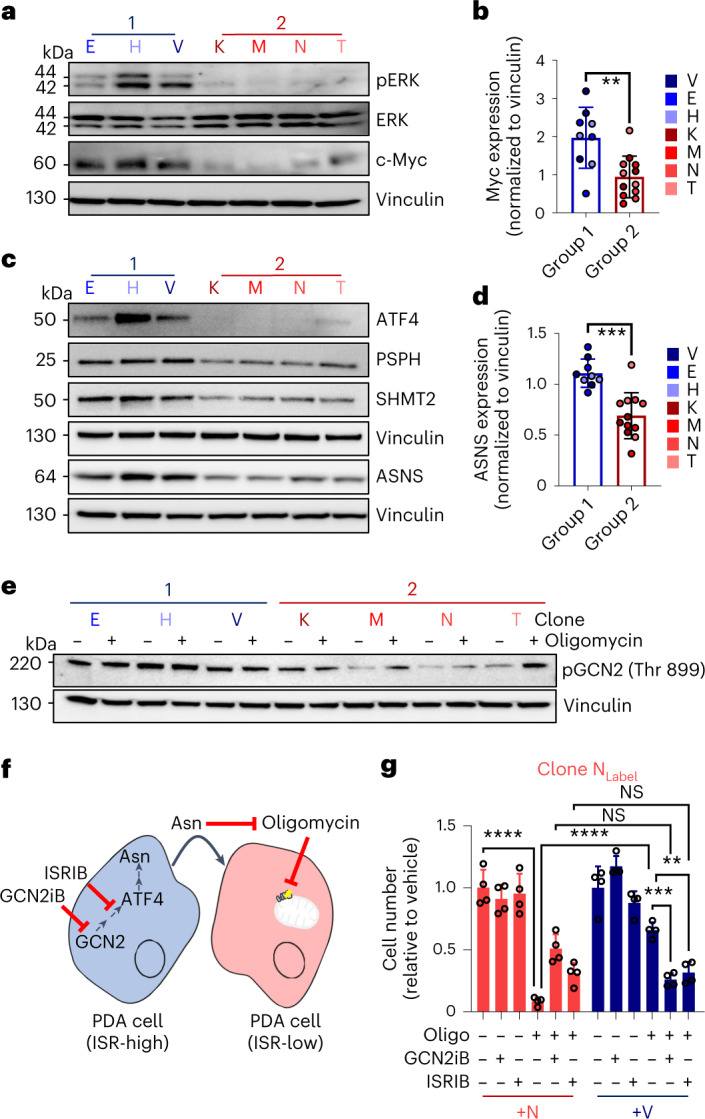
PDA clones engage different models of activation of the ISR. **a**, Immunoblot comparison of phospho-p44/42 MAPK Thr 202/Tyr 204 (pERK), total p44/42 MAPK (ERK), c-Myc and vinculin across group 1 (E, H and V) and group 2 (K, M, N and T) clonal populations. The image is representative of three independent experiments. **b**, Quantification of Myc expression across clonal groups normalized to vinculin loading controls (*n* = 3 blots per clone). **c**, Immunoblot comparison of ATF4, PSPH, SHMT2, ASNS and vinculin across group 1 (E, H and V) and group 2 (K, M, N and T) clonal populations. The image is representative of three independent experiments. **d**, Quantification of ASNS expression across clonal groups normalized to vinculin loading controls (*n* = 3 blots per clone). **e**, Expression of phospho-GCN2 T899 (pGCN2) and vinculin between group 1 (E, H and V) and group 2 (K, M, N and T) clonal populations treated with either vehicle or 1 nM oligomycin. **f**, Schematic representation of clonal cross-talk between ISR-high or ISR-low clones and key nodes to inhibit cross-talk. The image is representative of three replicates. **g**, Clonal line N was encoded with a fluorescent label, plated in direct coculture with unlabeled sensitive clone N or insensitive clone V and treated with oligomycin, ISRIB, GCN2iB, oligomycin and ISRIB, oligomycin and GCN2iB or vehicle (*n* = 3 replicates). Cells were counted, and endpoint data were plotted relative to vehicle; *P* ≤ 0.01, *** *P* ≤ 0.001 and **** *P* ≤ 0.0001 by two-tailed Mann–Whitney test (**b** and **d**) and one-way ANOVA with a Tukey post hoc test (**g**); *P* = 0.0043 (**b**), *P* = 0.0003 (**d**), +N versus +N + oligomycin *P* < 0.0001, +N + oligomycin versus +N + oligomycin *P* < 0.0001, +N + oligomycin + GCN2iB versus +V + oligomycin + GCN2iB *P* = 0.0844, +N + oligomycin + ISRIB versus +V + oligomycin + ISRIB *P* > 0.9999, +V + oligomycin versus +V + oligomycin + GCN2iB *P* = 0.0004 and +V + oligomycin versus +V + oligomycin + ISRIB *P* = 0.0038 (**g**). Source data

**Extended Data Fig. 6:**
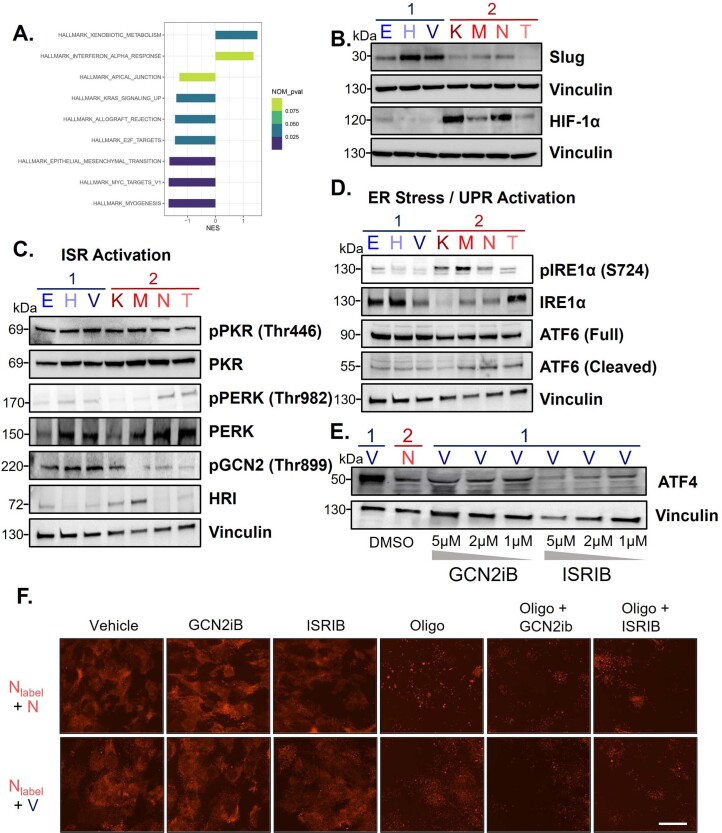
Transcriptional Programs and Stress Response in PDA Populations. **A**. Gene Set Enrichment Analysis (GSEA) of pathways differentially expressed between group 1 and group 2 clones. Negative NES scores indicate pathways enriched in group 1 and positive NES scores are pathways enriched in group 2. **B**. Immunoblot analysis of Slug and Hypoxia Inducible Factor 1 Subunit Alpha (HIF-1α) across group 1 clones (E,V,H) vs. group 2 (K,M,N,T). **C**. Immunoblot analysis of integrated stress response (ISR) activation proteins general control nonderepressible 2 (GCN2), protein kinase RNA-like ER kinase (PERK), protein kinase RNA-activated (PKR), and heme-regulated initiation factor 2 alpha kinase (HRI) across group 1 clones (E,V,H) vs. group 2 (K,M,N,T). **D**. Immunoblot analysis of ER stress and the unfolded-protein response (UPR) pathway activation proteins inositol-requiring enzyme 1 α (IRE1α) and activation transcription factor 6 (ATF6) across group 1 clones (E,V,H) vs. group 2 (K,M,N,T). **E**. Immunoblot analysis of ATF4 of insensitive clone V and sensitive clone N treated with DMSO, and insensitive clone V treated with 1, 2, and 5 µM of ISRIB or GCN2iB. **F**. Representative images from Fig. 4G of clonal line N was encoded with a fluorescent label, plated in direct co-culture with unlabeled sensitive clone N or insensitive clone V, and then treated with oligomycin, ISRIB, GCN2iB, oligomycin and ISRIB, oligomycin and GCN2iB, or vehicle. Scale bar = 1 mm. Source data

To determine what was driving the metabolic programs between the subtypes, we first queried a transcriptomic dataset from the clonal populations^30^. We observed that group 1 clones were enriched in pathways related to KRAS signaling, epithelial–mesenchymal transition and Myc signaling (Extended Data Fig. 6a,b). Indeed, assessing activation of these pathways by immunoblotting, we found that mitogen-activated protein kinase (MAPK) signaling downstream of KRAS, with corresponding activation of c-Myc, clearly segregated our clonal groups in accordance with their metabolism (Fig. 4a,b).

Previous studies have linked c-Myc expression/activity with the ISR^31,32^. Activating transcription factor 4 (ATF4) signaling downstream of the ISR supports amino acid homeostasis, suggesting a possible relationship with our extracellular metabolome profiling (Fig. 3a,b). Indeed, ATF4 expression clearly demarcated the groups, and this constitutively high expression in the group 1 clones was evident even with growth under nutrient-replete conditions (Fig. 4c,d). *ASNS* is among the most classical ATF4 target genes^33^. Consistent with both the expression of ATF4 and the release of asparagine from group 1 clones, these lines also express more ASNS than group 2 clones (Fig. 4c). Furthermore, we also observed that the ATF4 targets serine hydroxymethyltransferase 2 (SHMT2) and phosphoserine phosphatase (PSPH) are expressed at a higher level in the group 1 clonal populations.

ATF4 drives the ISR and is activated through numerous stresses, such as amino acid starvation by general control non-derepressible 2 (GCN2), endoplasmic reticulum (ER) stress through protein kinase RNA-like ER kinase (PERK), viral infection through protein kinase RNA-activated (PKR) and heme deprivation by heme-regulated initiation factor 2-α kinase (HRI)^34^. We performed immunoblotting for each of these pathways and observed that only GCN2 in consistently activated in the group 1 versus group 2 clones (Extended Data Fig. 6c). We also observed that group 1 clones are not engaged in a general stress response, as we did not find indications of increased ER stress or an unfolded protein response in the group 1 versus group 2 clones (Extended Data Fig. 6d). Conversely, we found inositol-requiring enzyme 1-α (IRE1α) and ATF6 activation to be enriched in the group 2 clones.

Interestingly, we found GCN2 to be both activated to a higher extent in the group 1 clonal population and non-responsive to further activation by oligomycin treatment (Fig. 4e). By contrast, the group 2 clones had a lower basal level of GCN2 phosphorylation and activated this pathway in response to treatment with oligomycin. Based on these collective data, group 1 clones exhibit constitutive activation of the ATF4 arm of the ISR, whereas the group 2 clones can induce this response when challenged. Next, to functionally validate the role of the ISR in mediating the rescue between insensitive and sensitive cells, we repeated our labeled coculture assays in the presence of pharmacological inhibitors of ISR pathway components (Fig. 4f). We first observed that an ISR inhibitor (ISRIB) or a direct GNC2 inhibitor (GCN2iB) are sufficient to reduce the expression of ATF4 in a constitutively active group 1 clone to similar levels as a group 2 clone (Extended Data Fig. 6e). Further, treatment with these compounds greatly impairs the rescue of proliferation of a sensitive clone treated with oligomycin mediated by the coculture with an insensitive clone (Fig. 4g and Extended Data Fig. 6f), confirming the functional importance of differential ISR expression between clonal lines and the role of GCN2 in driving this response.

### Differential ISR activation and function in human PDA

**Fig. 5:**
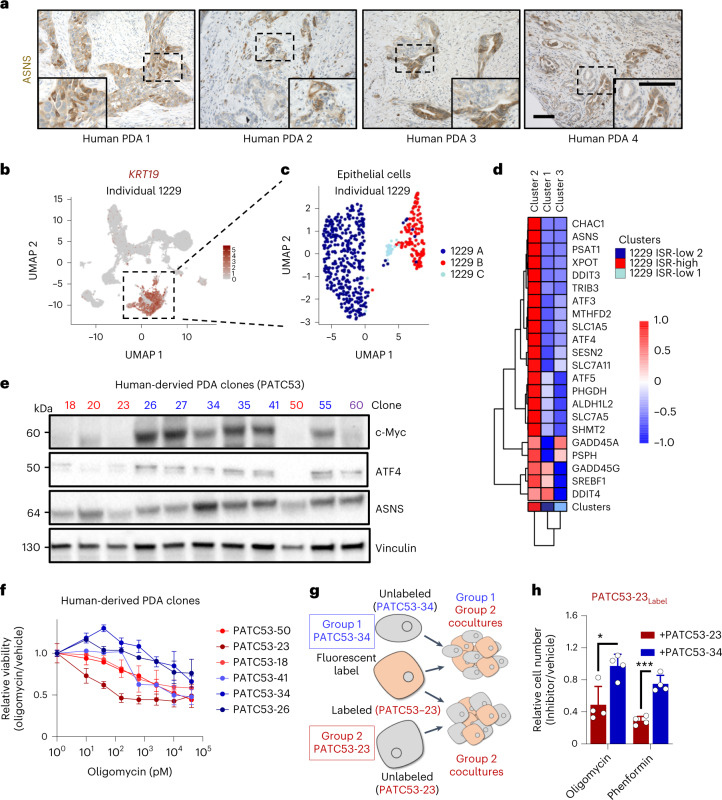
Human PDA tumors exhibit differential ISR activation. **a**, Immunostaining for ASNS in human PDA tissues; dashed boxes are magnified in insets; scale bars, 100 µm. **b**, UMAP representations of *KRT19* expression delineating the epithelial cell population identified from single-cell RNA analysis of a tumor biopsy of PDA individual 1229. **c**, UMAP representation of the three cell clusters within the epithelial population in **b**. **d**, Non-hierarchical clustering analysis of epithelial clusters across a set of ISR genes. **e**, Immunoblotting analysis of c-Myc, ATF4, ASNS and vinculin of clonal cells lines derived from the PATC53 human PDA tumor model; red, ISR low; blue, ISR high; purple, does not fit either category. The image is representative of three individual experiments. **f**, Dose–response of PATC53 clones to oligomycin; blue, insensitive clones; red, sensitive clones; *n* = 3 biological replicates per clone. **g**, Clonal line PATC53-23 was encoded with a fluorescent label and plated in direct coculture with unlabeled clones PATC53-23 or PATC53-34. **h**, Cell numbers of labeled PATC53-23 cocultures treated with 0.5 nM oligomycin or 125 µM phenformin relative to vehicle control (*n* = 3 biological replicates). Error bars are representative of mean ± s.d.; *P ≤ 0.05 and ***P ≤ 0.001 by two-tailed Student’s *t*-test; oligomycin *P* = 0.012 and phenformin *P* = 0.0002 (**h**). Source data

**Extended Data Fig. 7:**
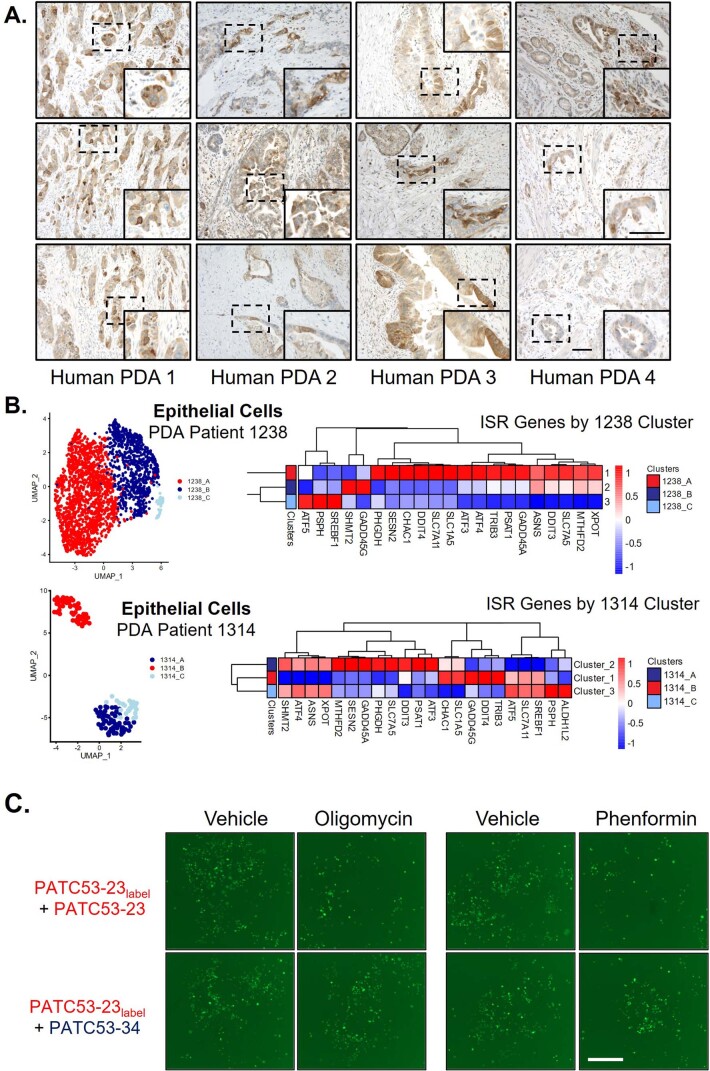
Interrogation of Human PDA Clonality. **A**. Immunostaining for ASNS in human PDA patient tissues, dashed boxes magnified in insets. Scale bars = 100 µm. **B**. Uniform Manifold Approximation and Projection (UMAP) representation of 3 epithelial cell clusters identified from single cell RNA analysis of a tumor biopsy of PDA Patients 1238 and 1314, with non-hierarchical clustering analysis of epithelial clusters across a set of integrated stress response (ISR) genes. **C**. Representative images from Fig. 5H. Clonal line PATC53-23 was encoded with a fluorescent label, plated in direct co-culture with unlabeled clones PATC53-23 or PATC53-34, then treated with 0.5 nM oligomycin or 125 µM phenformin relative to vehicle control. Scale bar = 1 mm.

To determine if the differential expression of ASNS was also found in human tumors, we immunostained human PDA samples for ASNS. As predicted from our mouse clonal lines, human PDA tumors revealed considerable intratumoral heterogeneity in ASNS expression (Fig. 5a and Extended Data Fig. 7a), with ASNS expression varying from absent to strong within the same ductal lesions. Next, we examined ISR and ATF4 pathway activity in single-cell RNA-sequencing datasets from human PDA^35^. Here, we observed the ATF4 gene signature to be strongly enriched within subgroups of malignant epithelial populations, a feature consistent across multiple human samples (Fig. 5b–d and Extended Data Fig. 7b).

To investigate if the differential expression of the ATF4 pathway was retained in isolated human PDA cells, we established a set of clonal cell lines derived from a human PDA tumor. Immunoblotting revealed that the expression levels of c-Myc, ATF4 and ASNS were correlated in 10 of 11 clones examined (Fig. 5e), with the one outlier expressing strong ATF4 and ASNS without concurrent c-Myc expression. Functionally validating a set of ASNS^hi^ versus ASNS^low^ lines, we found a trend toward low ASNS expression and sensitivity to oligomycin (Fig. 5f). Finally, we observed that the proliferation of a fluorescently labeled ATF4^low^ASNS^low^Myc^low^ (oligomycin-sensitive, group 2) human PDA clonal line can be rescued in the presence of oligomycin through coculture with an ATF4^hi^ASNS^hi^Myc^hi^ (oligomycin-resistant, group 1) human PDA clonal line (Fig. 5g,h and Extended Data Fig. 7c). Taken together, these data provide evidence for the existence of these same metabolic subtypes in human tumors.

### Asparagine rescue supports aspartate pools

**Fig. 6:**
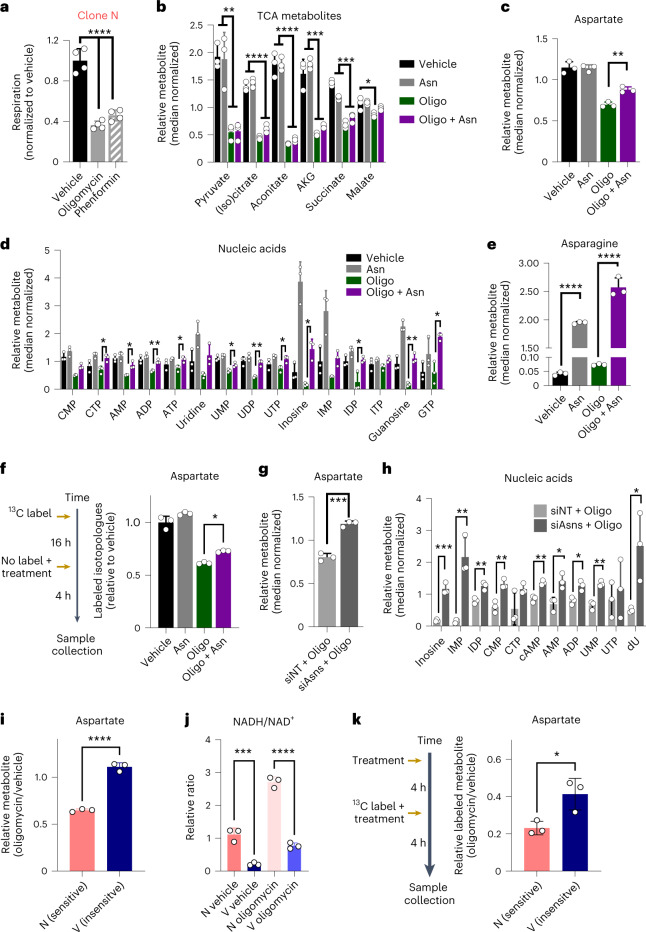
Asparagine rescues respiration inhibition by supporting aspartate pools. **a**, Oxygen consumption of clonal line N treated with vehicle, oligomycin or phenformin, as measured by a Seahorse metabolic flux analyzer (*n* = 4 samples per condition). **b**–**e**, Relative abundance of TCA cycle metabolites (**b**), aspartate (**c**), nucleotides (**d**) and asparagine (**e**) present in clone N cells treated with asparagine, oligomycin, asparagine + oligomycin or vehicle 4 h after treatment (*n* = 3 samples per condition). **f**, Labeled aspartate pools of clone N prelabeled with universally labeled ^13^C-glutamine and switched to medium containing unlabeled glutamine and treated with asparagine, oligomycin, asparagine + oligomycin or vehicle for 4 h (*n* = 3 samples per condition). **g**,**h**, Relative aspartate (**g**) and nucleotide levels (**h**) of siNT- or siAsns-transfected clone N cells treated with 1 nM oligomycin for 4 h (*n* = 3 samples per condition). **i**, Aspartate levels of oligomycin-sensitive clone N and oligomycin-insensitive clone V relative to vehicle 4 h after treatment (*n* = 3 samples per condition). **j**, Ratio of NADH/NAD^+^ of clone N and clone V treated for 4 h with oligomycin or vehicle (*n* = 3 samples per condition). **k**, Labeled aspartate pools of sensitive clone N and insensitive clone V were treated for 4 h with oligomycin or vehicle and switched to medium containing universally labeled ^13^C-glutamine and treated with oligomycin or vehicle for an additional 4 h (*n* = 3 samples per condition); *P ≤ 0.05, **P ≤ 0.01 and ****P ≤ 0.0001 by one-way ANOVA with a Tukey post hoc test (**a**–**f** and **j**) or two-tailed Student’s *t*-test (**g**–**i** and **k**); vehicle versus oligomycin *P* < 0.0001, vehicle versus phenformin *P* < 0.0001 (**a**); pyruvate: vehicle versus oligomycin *P* = 0.0017, vehicle versus oligomycin + asparagine *P* = 0.0019, asparagine versus oligomycin *P* = 0.0021 and asparagine versus oligomycin + asparagine *P* = 0.0023; (iso)citrate: vehicle versus oligomycin *P* < 0.0001, vehicle versus oligomycin + asparagine *P* < 0.0001, asparagine versus oligomycin *P* < 0.0001, asparagine versus oligomycin + asparagine *P* < 0.0001; aconitate: vehicle versus oligomycin *P* < 0.0001, vehicle versus oligomycin + asparagine *P* < 0.0001, asparagine versus oligomycin *P* < 0.0001, asparagine versus oligomycin + asparagine *P* < 0.0001; α-ketoglutarate (AKG): vehicle versus oligomycin *P* < 0.0001, vehicle versus oligomycin + asparagine *P* = 0.0002, asparagine versus oligomycin *P* < 0.0001, asparagine versus oligomycin + asparagine *P* < 0.0001; succinate: vehicle versus oligomycin *P* < 0.0001, vehicle versus oligomycin + asparagine *P* < 0.0001, asparagine versus oligomycin *P* < 0.0001, asparagine versus oligomycin + asparagine *P* = 0.0004; malate: vehicle versus oligomycin *P* = 0.0208 (**b**); oligomycin versus oligomycin + asparagine *P* = 0.0072 (**c**); oligomycin versus oligomycin + asparagine: CTP *P* = 0.0295, AMP *P* = 0.0301, ADP *P* = 0.0082, ATP *P* = 0.0449, UMP *P* = 0.0348, UDP *P* = 0.0035, UTP *P* = 0.0429, inosine *P* = 0.0276, IDP *P* = 0.0101, guanosine *P* = 0.0015, GTP *P* = 0.0147 (**d**); vehicle versus asparagine *P* < 0.0001, oligomycin versus oligomycin + asparagine *P* < 0.0001 (**e**); *P* = 0.0106 (**f**); *P* = 0.0003 (**g**); siNT + oligomycin versus siAsns + oligomycin inosine *P* = 0.0007, IMP *P* = 0.0045, IDP *P* = 0.006, CMP *P* = 0.0058, cAMP *P* = 0.0022, AMP *P* = 0.0171, ADP *P* = 0.013, UMP *P* = 0.0017, dU *P* = 0.0308 (**h**); N versus V *P* < 0.0001 (**i**); N vehicle versus V vehicle *P* = 0.0002, N oligomycin versus V oligomycin *P* < 0.0001 (**j**); *P* = 0.027 (**k**). Source data

**Extended Data Fig. 8:**
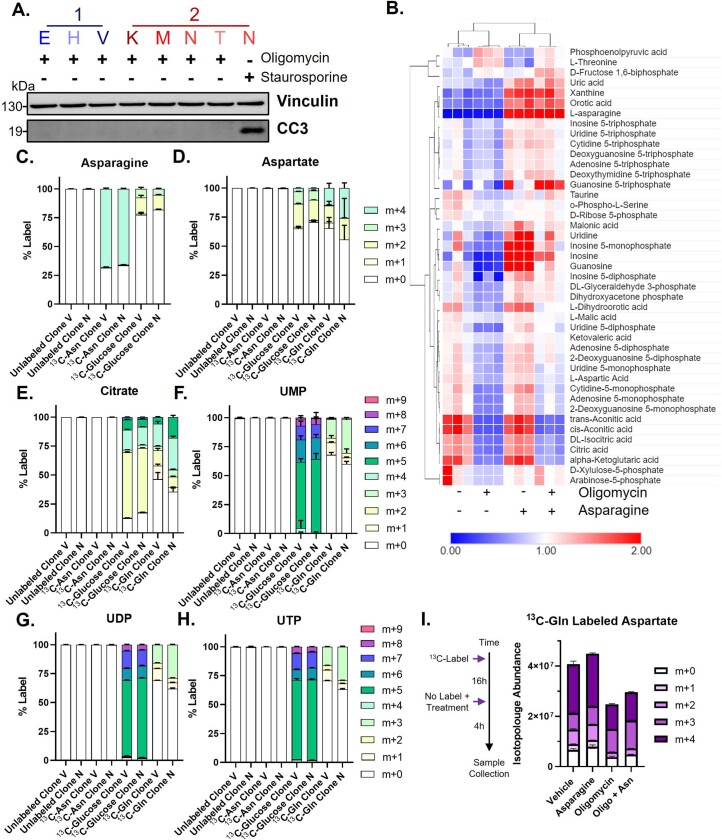
Impacts of Asparagine Rescue on Metabolism. **A**. Immunoblot analysis of Vinculin and Cleaved Caspase 3 across 1 nM treated oligomycin group 1 clones (E,V,H) vs. group 2 (K,M,N,T). Staurosporine treated clonal line N is included as a positive control. **B**. Non-hierarchical clustering heatmap of metabolites that differed between experimental conditions (p = 0.1) from clone N cells treated with asparagine, oligomycin, asparagine + oligomycin, or vehicle 4 hours post treatment (n = 3). **C-H**. Isotopic labeling patterns in clones N and V using media supplemented with universally-labeled ^13^C-asparagine (Asn), ^13^C-glucose, or ^13^C-glutamine, relative to unlabeled controls, for asparagine (**C**, n = 3), aspartate (**D**, n = 3), citrate (**E**, n = 3), or uridinylates (**F-H**, n = 3). **I**. Isotopologue abundance of aspartate from clone N pre-labeled with universally labeled ^13^C-glutamine, then switched to media containing unlabeled-glutamine and treated with asparagine, oligomycin, asparagine + oligomycin, or vehicle for 4 hours (n = 3). Error bars are mean ± SD. Source data

We next sought to determine how exogenous asparagine mediates rescue of mitochondrial inhibition. We first noted that concentrations of oligomycin and phenformin that can be rescued by asparagine supplementation impair, but do not abolish, respiration (Fig. 6a). Additionally, we also did not observe caspase 3 cleavage at these concentrations of mitochondrial inhibition, suggesting that the mechanism by which asparagine enables proliferation during impaired respiration is not through suppression of apoptosis, as in other systems^36^ (Extended Data Fig. 8a).

To determine how asparagine rescues impaired respiration, we then performed targeted metabolomics on cells treated with oligomycin in the presence or absence of asparagine (Extended Data Fig. 8b and Supplemental Table 3). Consistently, and in line with previous data using other mitochondrial poisons^29,37,38^, oligomycin treatment depleted TCA cycle intermediates (Fig. 6b) and associated branching metabolites, including aspartate (Fig. 6c). Aspartate is the biosynthetic precursor for de novo nucleotide biosynthesis, and nucleotide pools were similarly down after oligomycin treatment (Fig. 6d). The combination of asparagine with oligomycin treatment did not impact the pool size of TCA cycle intermediates (Fig. 6b). By contrast, asparagine treatment yielded a modest but significant increase in aspartate pools in the oligomycin-treated cells, a more notable increase in nucleic acid pools^27^ and an expected, yet pronounced, increase of asparagine levels (Fig. 6c–e).

In human and mouse cells, the enzymatic machinery to convert asparagine to aspartate does not exist^37^, which argues against asparagine contributing directly to aspartate pools. Indeed, using stable isotope tracing metabolomics, we demonstrated that asparagine carbon is not contributing to aspartate, TCA cycle intermediates or nucleotide pools (Extended Data Fig. 8c–h). Together, these data suggest that the impact of asparagine on proliferation may instead be to relieve the demand on aspartate pools required for asparagine biosynthesis. In other words, asparagine supplementation enables the limited aspartate, incurred by inhibition of respiration, to be used in amino acid synthesis and to make other rate-limiting intermediates, like nucleic acids.

To examine this directly, we performed a pulse–chase assay by introducing a universally labeled ^13^C-glutamine isotope overnight and challenging cells with oligomycin, with or without asparagine, in the absence of label for 4 h. Here, we found that that aspartate pools were robustly labeled (Extended Data Fig. 8i), and the label was retained in aspartate in the asparagine-treated samples during oligomycin challenge (Fig. 6f). To further test this model, we inhibited *Asns* by RNAi to decrease the asparagine synthesis pull on aspartate pools during stress, concurrent with 4 h of oligomycin treatment. In doing so, we observed an increase in aspartate, indicating that the demand for asparagine biosynthesis does indeed impact aspartate levels (Fig. 6g). Furthermore, this also permitted increases in nucleic acid pools at this time point (Fig. 6h).

**Extended Data Fig. 9:**
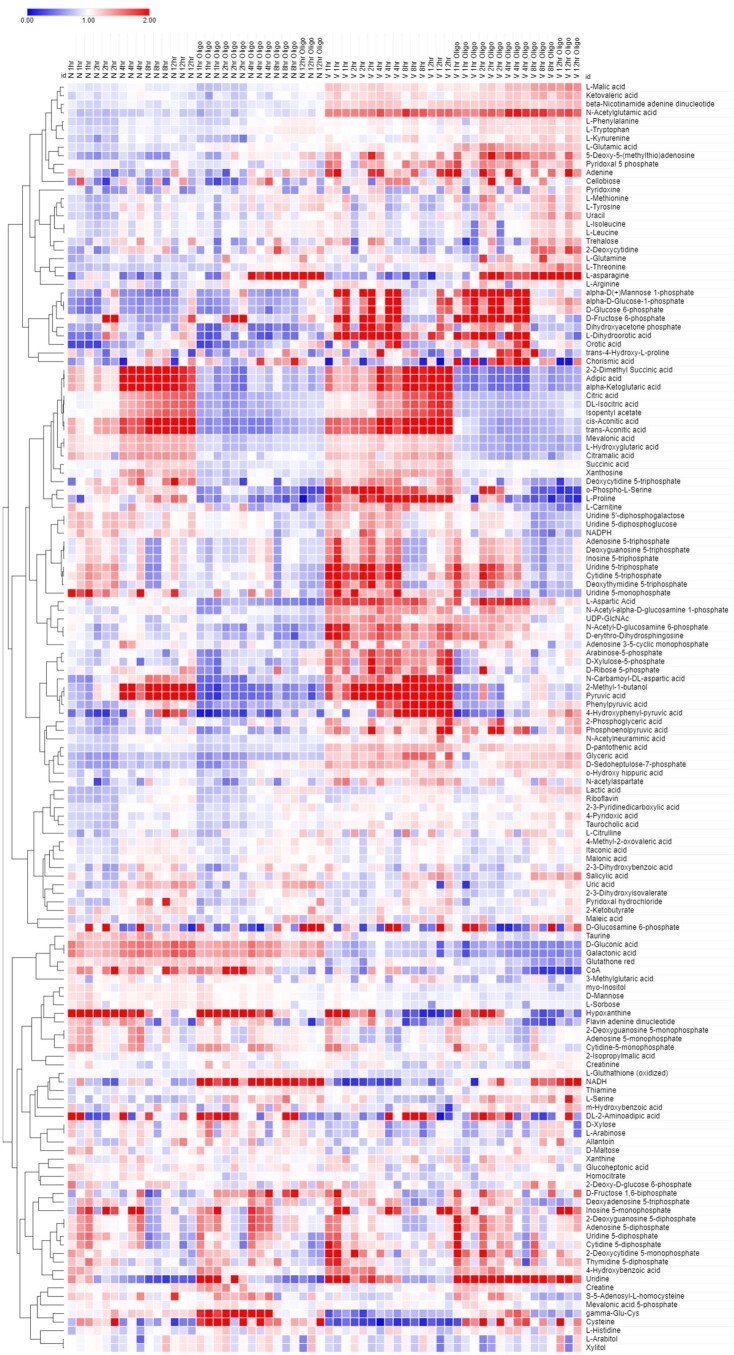
Time Course Metabolomics. Non-hierarchical clustering heatmap of metabolites that differed between experimental conditions (p = 0.1) from sensitive clone N or insensitive clone V cells treated with asparagine, oligomycin, asparagine + oligomycin, or vehicle and harvested at 1, 2, 4, 8, and 12 hours post treatment (n = 3).

**Extended Data Fig. 10:**
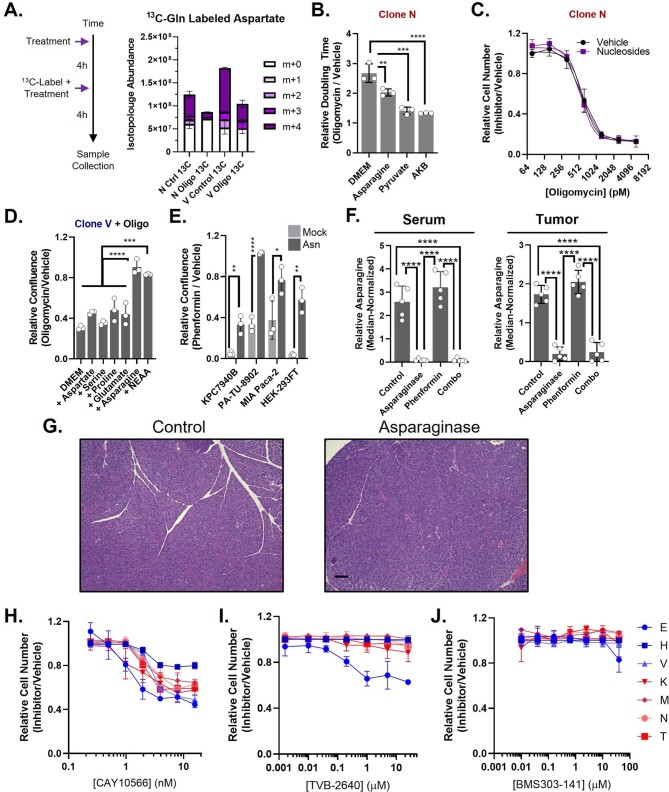
Asparagine Rescue and Tumor Study Controls. **A**. Isotopologue abundance of aspartate from sensitive clone N and insensitive clone V treated for 4 hours with oligomycin or vehicle, then switched to media containing with universally labeled ^13^C-glutamine and treated with oligomycin or vehicle for an additional 4 hours (n = 3). **B**. Relative doubling time of clone N cells treated with 1 nM oligomycin with or without the addition of exogenous asparagine, pyruvate, or alpha-ketobutyrate (AKB) (n = 3). **C**. Dose response curve of clone N cells treated with oligomycin in the presence of absence of 100 µM exogenous nucleosides (n = 3). **D**. Plate confluence at endpoint of respiration-inhibition insensitive clone V treated with 1.5 nM oligomycin in the presence of absence of media containing all NEAAs or individual NEAAs (n = 3). **E**. Plate confluence at endpoint of cell lines treated with phenformin with or without 100 µM exogenous asparagine; KPC7940B and HEK-293FT 150 µM, PA-TU-8902 and MIA PaCa-2 37.5 µM (n = 3). **F**. Relative metabolite levels of asparagine in the serum and tumor lysate of mice bearing orthotopic KPC-7940B treated with vehicle, phenformin, asparaginase, or phenformin + asparaginase (n = 5 each). **G**. H&E staining of pancreas from WT C57BL/6 J treated with either vehicle or asparaginase. **H-J**. Dose response curves across clonal lines of the Stearoyl-CoA desaturase-1 (SCD) inhibitor CAY10566 (**H**, n = 3), Fatty Acid Synthase (FASN) inhibitor TVB-2640 (**I**, n = 3), and ATP citrate lyase inhibitor (ACLY) BMS303-141 (**J**, n = 3). Scale bar = 100 µm. Error bars are mean ± SD, * *P* ≤ 0.05; ** P ≤ 0.01; *** P ≤ 0.001; **** P ≤ 0.0001 by two-tail student’s t test (**E**) or one-way Anova with Tukey post hoc (**B,D,F**). Source data

Given the central role of aspartate revealed through these experiments, we postulated that aspartate metabolism will present a key difference between our group 1 and group 2 clonal populations. To examine this, we performed a metabolomics time course analysis of group 1 and group 2 clones treated with oligomycin (Extended Data Fig. 9 and Supplemental Table 4). Indeed, at the same 4-h time point as the previous experiment, we observed that the insensitive clone maintained higher relative aspartate pools when challenged with oligomycin than the sensitive clone (Fig. 6i). The insensitive clone also presented with a lower NADH/NAD^+^ ratio (Fig. 6j), which was similarly evident following oligomycin treatment, suggesting an increased capacity to maintain aspartate biosynthesis under both conditions. Indeed, we found that universally labeled ^13^C-glutamine provided during an oligomycin challenge is still incorporated into aspartate pools, and this occurs to a larger extent than vehicle-treated controls in an insensitive clone versus a sensitive clone (Fig. 6k and Extended Data Fig. 10a). Together, these data suggest that the relative insensitivity of group 1 clones to mitochondrial inhibitors may be in part mediated by lower basal reductive stress and a higher reservoir of aspartate, providing a buffer against depletion during a period of impaired respiration.

Lastly, consistent with both our model and previous work^29^, the electron acceptors pyruvate and α-ketobutyrate rescue proliferation from impaired respiration (Extended Data Fig. 10b). These rescue agents act upstream of asparagine by relieving NADH reductive stress imparted by electron transport chain inhibition and thereby facilitate TCA cycle flux and aspartate biosynthesis. In line with this more direct role, these rescue agents more effectively reverse the proliferative defects imposed by inhibition of respiration. Nucleotide supplementation alone was not sufficient to rescue proliferation (Extended Data Fig. 10c), suggesting additional roles for asparagine. Collectively, these data indicate that asparagine rescues the proliferative defects imparted by mitochondrial inhibition, at least in part, by decreasing the demand on aspartate.

### Asparagine rescues limited respiration across cell lines

**Fig. 7:**
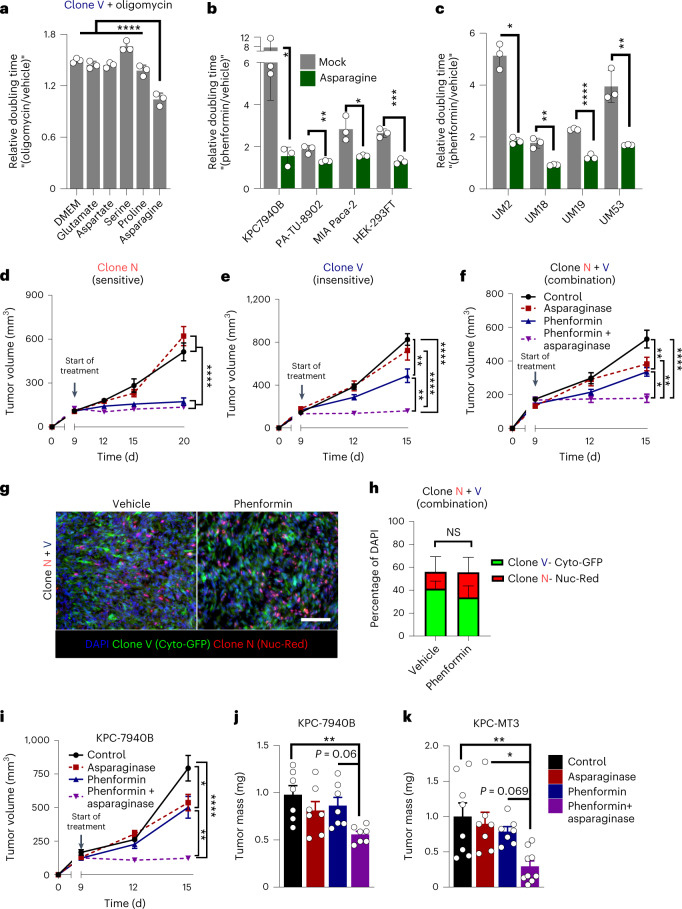
Asparagine rescues the inhibition of respiration in diverse systems and is an exploitable vulnerability in pancreatic cancer. **a**, Doubling times of respiration inhibition-insensitive clone V treated with 1.5 nM oligomycin in the presence or absence of medium containing all NEAAs or individual NEAAs (*n* = 3 samples per condition). **b**, Relative doubling times of cell lines treated with phenformin with or without 100 µM exogenous asparagine; KPC7940B and HEK-293FT 150 µM, PA-TU-8902 and MIA PaCa-2 37.5 µM; *n* = 3 samples per condition. **c**, Human-derived PDA lines UM2, UM18, UM19 and UM53 treated with phenformin in the presence or absence of 100 µM exogenous asparagine (*n* = 3 samples per condition). **d**–**f**, Athymic nude mice were implanted subcutaneously with clone N (**d**), clone V (**e**) or a combination of clone N and V (**f**), and tumors were allowed to establish for 9 d. Mice were then treated with asparaginase, phenformin, asparaginase + phenformin or vehicle until collection (*n* = 10 tumors per treatment.). **g**, Representative images (one of three) of frozen sections of vehicle- or phenformin-treated tumors established from co-injection of nuclear red (Nuc-Red)-labeled clone N and cytoplasmic GFP (Cyto-GFP)-labeled clone V; scale bar, 90 µm. **h**, Quantification of the respective fluorescent labels from **g** (*n* = 3 images per treatment group). **i**, C57BL/6J mice were implanted subcutaneously with syngeneic mouse KPC7940B PDA cells, and tumors were allowed to establish for 9 d. Mice were then treated with asparaginase, phenformin, asparaginase + phenformin or vehicle until collection (*n* = 10 tumors per treatment group). **j**,**k**, Final tumor mass from C57BL/6J mice implanted orthotopically with KPC-7490B (**j**; *n* = 7 per treatment group) or KPC-MT3 (**k**; *n* = 8 vehicle, *n* = 7 asparaginase, *n* = 7 phenformin and *n* = 9 phenformin + asparaginase tumors per treatment group) syngeneic mouse PDA cells and treated 14 d after establishment with asparaginase, phenformin, asparaginase + phenformin or vehicle until collection 10 d later. Error bars represent mean ± s.d. (**a**–**c** and **h**) and mean ± s.e.m. (**d**–**f** and **i**–**k**); *P ≤ 0.05, **P ≤ 0.01, ***P ≤ 0.001 and ****P ≤ 0.0001 by one-way ANOVA with a Tukey post hoc test (**a**, **d**–**f** and **i**–**k**) or a two-tailed Student’s *t*-test (**b** and **h**); DMEM versus asparagine *P* < 0.0001, asparagine versus glutamate *P* < 0.0001, asparagine versus aspartate *P* < 0.0001, asparagine versus serine *P* < 0.0001, asparagine versus proline *P* < 0.0001 (**a**); mock versus asparagine KPC7940B *P* = 0.0377, HEK-293FT *P* = 0.0006, MIA PaCa-2 *P* = 0.0204, PA-TU-8902 *P* = 0.008 (**b**); mock versus asparagine UM2 *P* = 0.0377, UM18 *P* = 0.0017, UM19 *P* < 0.0001, UM53 *P* = 0.0032 (**c**); control versus phenformin *P* < 0.0001, control versus phenformin + asparaginase *P* < 0.0001, asparaginase versus phenformin *P* < 0.0001, asparaginase versus phenformin + asparaginase *P* < 0.0001 (**d**); control versus phenformin *P* = 0.0021, control versus phenformin + asparaginase *P* < 0.0001, asparaginase versus phenformin + asparaginase *P* < 0.0001, phenformin versus phenformin + asparaginase *P* = 0.0029 (**e**); control versus phenformin *P* = 0.0043, control versus phenformin + asparaginase *P* < 0.0001, asparaginase versus phenformin + asparaginase *P* = 0.0028, phenformin versus phenformin + asparaginase *P* = 0.028 (**f**); Nuc-Red vehicle versus Nuc-Red phenformin *P* = 0.5435 (**h**); control versus phenformin *P* = 0.0268, control versus phenformin + asparaginase *P* < 0.0001, phenformin versus phenformin + asparaginase *P* = 0.002 (**i**); control versus phenformin + asparaginase *P* = 0.007, asparaginase versus phenformin + asparaginase *P* = 0.1573, phenformin versus phenformin + asparaginase *P* = 0.0647 (**j**); control versus phenformin + asparaginase *P* = 0.0036, asparaginase versus phenformin + asparaginase *P* = 0.0193, phenformin versus phenformin + asparaginase *P* = 0.0698 (**k**). Source data

Group 1 clones are less sensitive to growth inhibition by oligomycin (Fig. 1e). However, at higher oligomycin concentrations, the proliferation of group 1 clones can be similarly impaired. More importantly, this can be reversed by exogenous asparagine supplementation (Fig. 7a and Extended Data Fig. 10d). This suggests that asparagine rescue of respiration is not limited to populations of cells highly sensitive to mitochondrial inhibition. In support of this, we determined that asparagine can also rescue phenformin-mediated inhibition in polyclonal mouse PDA cells, human pancreatic cancer lines and non-cancerous HEK-293FT cells (Fig. 7b and Extended Data Fig. 10e). Importantly, we also found this to be true in a panel of low-passage human-derived PDA cell lines (Fig. 7c).

### Asparagine depletion enhances mitochondrial inhibitors

Our in vitro studies demonstrated that exogenous asparagine, derived from group 1 clones or supplemented in the medium, can enable cellular proliferation when respiration is inhibited. In extending this work to in vivo models, we hypothesized that the depletion of extracellular asparagine would function to sensitize tumors to mitochondrial inhibition^27^. To test this, we first used our clonal system. As expected, we observed that growth of tumors from a sensitive group 2 clone was markedly reduced by treatment with phenformin (Fig. 7d). By contrast, tumors from a group 1 clone were less responsive to phenformin (Fig. 7e). The group 1 cells appeared to protect the group 2 cells from phenformin when co-injected in a one-to-one ratio (Fig. 7f). To confirm that this was indeed the case, we assessed the representation of labeled clonal lines and did not observe a decrease in the number of sensitive group 2 clone cells in phenformin-treated tumors relative to vehicle-treated tumors (Fig. 7g,h). We then combined phenformin with PEGylated l-asparaginase, a protein therapeutic that degrades extracellular asparagine^39^. This combination halted tumor growth in both the single clone and co-injection tumor models (Fig. 7e,f).

To examine this in a more physiological context of heterogenous disease, as would be found in humans, we established syngeneic tumor allografts in immunocompetent mice using two KPC transplant models of pancreatic cancer. These were then treated with PEGylated l-asparaginase, phenformin or a combination. Asparagine was robustly depleted both systemically and within orthotopic tumors using this treatment strategy (Extended Data Fig. 10f). Of note, asparaginase treatment did not result in pancreatitis pathology, as seen in some individuals with leukemia^40^ (Extended Data Fig. 10g), and we did not find any clonal differences in response to lipogenic inhibitors (Extended Data Fig. 10h–j). Most importantly, large reductions in tumor growth were observed across several pancreatic cancer models when treated with the combination of phenformin and PEGylated l-asparaginase (Fig. 7i–k).

## Discussion

The concept of metabolic heterogeneity in cancer is complex and has been described for (1) different mutations in the same tumor type^41,42^, (2) the same driving oncogene in different tumor types^43^, (3) the location of the same tumor cells when seeded at different sites in the body^28^, (4) regional heterogeneity within the same tumors^25,44,45^, (5) metabolite consumption and inhibitor sensitivity^8,46,47^ and (6) tumor stage, for example, metastatic versus primary tumor cells^48,49^. In pancreatic cancer, a seminal study identified three metabolic subtypes by profiling metabolic properties and dependencies. These include the slow proliferating, glycolytic and lipogenic subtypes^8^. While we noted a sensitivity toward glycolytic inhibition and increased lactate production in group 1 clones, we did not identify differential lipogenic metabolism (Extended Data Fig. 10h–j) nor were significant differences in growth rate observed between our clonal subtypes. Other recent studies in PDA have also illustrated how small subpopulations of cancer cells that survive inhibition of mutant KRAS signaling or are capable of anchorage-independent growth demonstrate enhanced utilization of mitochondrial oxidative phosphorylation relative to bulk tumor cells^50,51^. Our data build on these collective observations by demonstrating that metabolic complexity is present at baseline within clonal populations derived from the same tumors with a stable genotype.

Indeed, we identify and describe two different metabolic subtypes within mouse and human PDA tumors marked by differential activation of ATF4 by the GCN2 arm of the ISR. Group 2 clones exhibit a more classical GCN2–ATF4 pathway activation in response to mitochondrial stress. By contrast, the group 1 clones have constitutively elevated GCN2–ATF4 signaling and expression of classical ATF4 target genes, including *ASNS*. This leads to constitutive production and release of asparagine through mass action. We then demonstrate that this asparagine can be captured and used by group 2 clones to support proliferation when respiration is inhibited.

Despite the considerable potential for metabolic complexity in tumors, our data still demonstrate that it is possible to target metabolism in cancer cells. Indeed, tumor metabolism has been an attractive target in PDA given the limited impact of conventional chemotherapy and immunotherapy on survival^4^. Phenformin was recently identified as the most effective metabolic inhibitor in a screen of PDA human-derived xenografts^52^. It is currently being examined clinically in other cancer types (NCT03026517), and our data show that this can be potentiated by asparagine depletion using asparaginase. Asparaginase is a widely used clinical agent for the treatment of blood cancers^53^, and it is in clinical trials for pancreatic cancer combined with chemotherapy (NCT02195180)^54^. Furthermore, there are also more specific and potent mitochondrial inhibitors currently being deployed in the clinic^55^, including in pancreatic cancer (NCT03291938), and these could similarly benefit from combination with asparaginase.

Therapeutic application of mitochondrial inhibitors in PDA may have additional avenues of action. For example, we have shown that immunosuppressive tumor-associated macrophages in pancreatic cancer primarily use mitochondrial metabolism^13^. Accordingly, the use of phenformin and other mitochondria-targeting compounds might also serve to sensitize pancreatic tumors to immune-based therapies, similar to that seen targeting glutamine metabolism and the hexosamine biosynthetic pathway^55,56^. Moreover, efforts to classify targetable growth-promoting cross-talk interactions between neoplastic and non-neoplastic cells in the tumor are likely to be enhanced by the growing body of data coming out of single-cell analyses. Together, these will allow us to continue to find useful new targets for these difficult to treat diseases.

These findings also add to a growing body of literature describing the asparagine-mediated cellular processes that promote proliferation under metabolic stress, such as rescuing apoptosis in the absence of glutamine^36,57^ and mediating mTOR signaling and anabolism by promoting amino acid exchange^27,58^. Furthermore, while the role of aspartate in supporting proliferation in the absence of respiration is now well established^29,38^, its limited cell permeability requires supraphysiological concentrations for aspartate to serve this role in pancreatic cancer (Fig. 2e). This likely results from the absence of an appropriate aspartate transporter, typically restricted to neuronal tissues but seen in some other cancers^59,60^. Given that asparagine is capable of promoting proliferation under mitochondrial stress, and at physiological concentrations^28^, there is likely a combination of these identified and further unidentified roles for asparagine, the exploration of which will provide exciting new avenues of study.

## Methods

The experiments in this study were performed in compliance with the Institutional Biosafety Committees and Institutional Animal Care and Use Committees of the University of Michigan (IBC00001639 and PRO00008877, C.A.L.) and the University of California, Irvine (BUA-R315 and AUP-20-102, C.J.H.).

### Cell culture

The clonal cell lines described (E, H, V, K, M, N and T) were isolated from pancreatic tumors from KPC mice and have been previously described^30^. Clonal lines 6419c5, 6694c2, 2838c3 and 6499c4 were derived from KPC mice and have also been previously described^24^. KPC7940B cells were a gift from G. Beatty (University of Pennsylvania). KPC-MT3 cells were a gift from D. Tuveson (Cold Spring Harbor Laboratory). PATC53 cells were obtained from ATCC. Cells were maintained in high-glucose DMEM (Gibco) supplemented with 10% fetal bovine serum (Corning) and routinely tested for mycoplasma contamination using MycoAlert Plus (Lonza). DMSO, oligomycin, phenformin and aminooxyacetic acid were obtained from Sigma, *trans*-ISRIB was obtained from Cayman Chemical and GCN2iB was obtained from MedChemExpress. IACS-10759 was obtained from Selleck Chem. KPC clonal lines were genotyped for *Kras*^G12D^ recombination using primer sets from Jackson Laboratories.

### Metabolite sample preparation

Intracellular metabolite fractions were prepared from cells grown in six-well plates (Corning) that were lysed with cold (−80 °C) 80% methanol and clarified by centrifugation. Metabolite levels of intercellular fractions were normalized to the protein content of a parallel sample, and all samples were lyophilized via speed vac after clarification by centrifugation. Media samples were prepared by collecting 200 µl of conditioned medium or basal medium and adding 800 µl of 100% methanol; the resultant solution was clarified by centrifugation and dried via speed vac. Dried metabolite pellets from cells or media were resuspended in 35 μl of a 50:50 HPLC-grade methanol:water mixture for metabolomics analysis.

### Metabolomics

Steady-state metabolomics analyses were conducted by running samples on either an Agilent 1290 UHPLC-6490 triple quadrupole (QqQ) MS/MS system or an Agilent 1290 Infinity II UHPLC-6470 QqQ MS/MS system. Stable isotopolog tracing analysis was performed on an Agilent 1290 Infinity II UHPLC and 6545B Accurate-Mass Quadrupole Time-of-Flight (MS Q-TOF) LC–MS.

#### 6490 parameters

For negative ion acquisition, a Waters Acquity UPLC BEH amide column (2.1 × 100 mm, 1.7 µm) was used, with mobile phase A consisting of 20 mM ammonium acetate (pH 9.6) in water and mobile phase B consisting of acetonitrile. The following gradient was used: mobile phase B was held at 85% for 1 min, increased to 65% in 12 min and to 40% in 15 min and held for 5 min before going to the initial condition and holding for 10 min. For positive ion acquisition, a Waters Acquity UPLC BEH TSS C18 column (2.1 × 100 mm, 1.7 µm) was used, with mobile phase A consisting of 0.5 mM ammonium fluoride and 0.1% formic acid in water and mobile phase B consisting of 0.1% formic acid in acetonitrile. The following gradient was used: mobile phase B was held at 1% for 1.5 min, increased to 80% in 15 min, increased to 99% in 17 min and held for 2 min before going to the initial condition and holding for 10 min. The column was kept at 40 °C, and 3 µl of sample was injected into the LC–MS/MS with a flow rate of 0.2 ml min^–1^. Tuning and calibration of the QqQ was achieved through Agilent electrospray ionization (ESI) low concentration tuning mix.

Optimization was performed on the 6490 QqQ in negative or positive mode individually for each of the 220 standard compounds to get the best fragment ion and other MS parameters for each standard. Retention time for each standard of the 220 standards was measured from pure standard solution or a mix standard solution. The LC–MS/MS method was created with dynamic multiple reaction monitoring ((d)MRMs) with retention times, retention time windows and MRMs of all the 220 standard compounds.

In both acquisition modes, key parameters of Agilent Jet Stream ESI were a gas temperature of 275 °C, gas flow of 14 liters min^–1^, nebulizer at 20 psi, sheath gas heater at 250 °C, sheath gas flow of 11 liters min^–1^ and capillary voltage of 3,000 V. For negative-mode MS, delta EMV was 350 V, cycle time was 500 ms and the cell accelerator voltage was 4 V. For positive acquisition mode MS, the delta EMV was set at 200 V with no change in cycle time and cell accelerator voltage.

#### 6470 parameters

The Agilent QqQ 6470 LC–MS system consists of a 1290 Infinity II LC flexible pump (Quaternary Pump), 1290 Infinity II multisampler, 1290 Infinity II multicolumn thermostat with a six-port valve and a 6470 QqQ mass spectrometer. Agilent Masshunter Workstation Software LC–MS Data Acquisition for 6400 Series QqQ MS with version B.08.02 was used for compound optimization and sample data acquisition.

Solvent A was 97% water and 3% methanol, 15 mM acetic acid and 10 mM tributylamine at a pH of 5. Solvent C was 15 mM acetic acid and 10 mM tributylamine in methanol. Washing solvent D was acetonitrile. The LC system seal-washing solvent was 90% water and 10% isopropanol; the needle wash solvent was 75% methanol and 25% water. The following associated chemicals were obtained: GC-grade tributylamine 99% (Acros Organics), LC–MS-grade acetic acid Optima (Fisher Chemical), InfinityLab Deactivator additive, ESI-L low concentration tuning mix (Agilent Technologies), LC–MS-grade solvents of water and acetonitrile, methanol (Millipore) and isopropanol (Fisher Chemical).

For separation, an Agilent ZORBAX RRHD Extend C18 column (2.1 × 150 mm, 1.8 µm) and ZORBAX Extend Fast Guards for UHPLC were used. The LC gradient profile was at 0.25 ml min^–1^, 0–2.5 min, 100% A; 7.5 min, 80% A and 20% C; 13 min 55% A and 45% C; 20 min 1% A and 99% C; 24 min 1% A and 99% C; 24.05 min 1% A and 99% D; 27 min 1% A and 99% D; at 0.8 ml min^–1^, 27.5–31.35 min, 1% A and 99% D; at 0.6 ml min^–1^, 31.50 min, 1% A and 99% D; at 0.4 ml min^–1^, 32.25–39.9 min, 100% A; at 0.25 ml min^–1^, 40 min, 100% A. The column temperature was kept at 35 °C, and samples were at 4 °C. The injection volume was 2 µl.

The 6470 QqQ was calibrated with ESI-L low concentration tuning mix. The following source parameters were used: gas temperature of 150 °C, gas flow of 10 liters min^–1^, nebulizer at 45 psi, sheath gas temperature of 325 °C, sheath gas flow of 12 liters min^–1^, capillary voltage of −2,000 V and delta EMV of −200 V. Dynamic MRM scan type was used with a 0.07-min peak width and acquisition time of 24 min. Delta retention time of ±1 min, fragmentor of 40 eV and cell accelerator of 5 eV were incorporated in the method.

The MassHunter Metabolomics Dynamic MRM Database and Method was used for target identification. Key parameters of the Agilent Jet Stream ESI were gas temperature of 150 °C, gas flow of 13 liters min^–1^, nebulizer at 45 psi, sheath gas temperature of 325 °C, sheath gas flow of 12 liters min^–1^, capillary voltage of 2,000 V, nozzle at 500 V and detector delta EMV at 200 V.

#### 6545 parameters

Chromatography followed the same method and setup as the 1290 Infinity II LC method listed above for the 6740 parameters. MS was performed with an Agilent G6545B MS Q-TOF with Dual AJD ESI sources in Profile Mode. The following instrument parameters were used: gas temperature of 250 °C, gas flow of 13 liters min^–1^, nebulizer at 35 psi, sheath gas temperature at 325 °C and sheath gas flow of 12 liters min^–1^. The following scan source parameters were used: negative polarity, collision energy 0, VCap 3,500 V, nozzle voltage 1,000 V, fragmentor 130 V, Skimmer1 60 V and OctopoleRFPeak 750 V. Auto calibration with reference masses of negative ions of 980.01637500 and 59.01390000 Da from the Agilent Reference Mix Solution were used. Calibration of MS Q-TOF 6545B with Agilent TOF calibration standard solution with a 1:100 dilution with 90% acetonitrile and 10% water solvent mixture was performed.

The QqQ data were preprocessed with Agilent MassHunter Workstation Quantitative Analysis software (B0700). Additional analyses were postprocessed for further quality control in the programming language R. In addition to preprocessing normalization by sample protein concentration, post-run samples were further normalized by the total intensity of all metabolites up to, but not exceeding, 20% variation. Samples with greater variation were excluded from further analysis. Finally, each metabolite abundance level in each sample was divided by the median of all abundance levels across all samples for proper comparisons, statistical analyses and visualizations among metabolites. Data were assessed by a two-tailed *t*-test with a significance threshold level of 0.05.

Heat maps were generated and data were clustered using a one minus Pearson correlation and average linkage across rows and columns on the Morpheus Matrix Visualization and Analysis tool (https://software.broadinstitute.org/morpheus).

The stable isotopolog tracing analysis was performed in Agilent MassHunter Profinder (10.0). Metabolites were verified by independent standards and used to create a database of retention times in Agilent MassHunter PCDL Manager (B0700) along with the Agilent 6470 dMRM database of the 220-metabolite library, which includes retention time and molecular formula information. The metabolite library was applied to the raw data through the isotopolog wizard to determine isotope distribution data.

### Lactate production measurement

Lactate measurements were performed using the lactate fluorescence assay kit (Biovision, K607). Assays were performed according to the manufacturer’s instructions. Lactate levels were measured using a SpectraMax M3 microplate reader (Molecular Devices).

### Citrate synthase assay

The citrate synthase assay followed a slightly modified protocol from http://wiki.oroboros.at/index.php/MiPNet17.04_CitrateSynthase. Briefly, a small aliquot of cells in suspension (72,000 cells) was placed in a solution containing 300 mM Tris (pH 8.1; T1503, Sigma), 0.25% Triton X-100 (T8532, Sigma), 0.31 mM acetyl-CoA (A2181, Sigma), 0.1 mM DTNB (D218200, Sigma) and 0.5 mM oxaloacetate (04126, Sigma) at 30 °C. Immediately, the increase in absorbance was measured at a wavelength of 412 nm. The rate of increase is directly proportional to the citrate synthase activity (extinction coefficient of TNB at 412 nm and pH 8.1 is 13.6 mM^–1^ cm^–1^). The assays were performed in triplicate, and the clones were measured on four separate time points from different culture plates.

### Seahorse metabolic flux assay

Seahorse assays were performed using a XF-96 Extracellular Flux Analyzer (Agilent). The day before the assay, sensor cartridges were preincubated in distilled water overnight, and cells were seeded at 12,000–15,000 cells per well. The next day, cells were washed, and medium was switched to Seahorse DMEM medium (pH 7.4; Agilent, 103575) supplemented with 25 mM glucose (Agilent, 103577) and 4 mM glutamine (Agilent, 103579). Cells were allowed to equilibrate for 0.5–1 h in a non-CO_2_ incubator at 37 °C before the assay. Sensor cartridges were hydrated in in XF calibrant (Agilent, 100840) for 1 h in a non-CO_2_ incubator at 37 °C. Hydrated cartridges were loaded with oligomycin (2 μM), FCCP (0.5–2 μM) and rotenone/antimycin A (1 μM) for the mitostress test or rotenone/antimycin A (1 μM) and 2-deoxyglucose (50 mM) for the glycolytic rate assay. After the assay, measurements were adjusted/normalized based on cell density using CyQuant (Invitrogen). The metabolic phenotype was determined based on basal oxygen consumption rate and extracellular acidification rate measurements (that is, before inhibitor treatment). Spare respiratory capacity was determined by subtracting basal oxygen consumption rate from maximal oxygen consumption rate measurements.

### NAD(P)H FLIM imaging

FLIM imaging was performed as previously described^61^. Briefly, 5 × 10^4^ cells were seeded in eight-well chamber cover glass (CellVis). After 48 h, the cells were subjected to Phasor FLIM imaging. The imaging plane was chosen close to the coverslip, and the fraction was evaluated in the area below the nucleus.

### Mitochondrial staining

Cells were stained with MitoTracker Red (Thermo Fisher), MitoTracker Green (Thermo Fisher) or TMRM (Thermo Fisher) for 30 min and detached and assessed via flow cytometry on an ACEA NovoCyte Quanteon using NovoExpress flow cytometry software (version 1.6.0); representative gating is shown in Supplemental Fig. 1. MitoTracker Red images were obtained on Amnis ImageStream X Mk II. Histograms were generated using FloJo (v10.8) software, and an equal number of cells was plotted per peak.

### Cell viability/proliferation assays

One thousand cells were seeded per well in a 96-well plate and placed on an orbital rocker for 20 min to ensure even spreading. Cells were equilibrated overnight, and compounds were added the next day. FX11, oligomycin, aminooxyacetic acid (AOA) and phenformin plates were read 4 d later on the IncuCyte S3 using phase object confluence as a readout. The half-maximum inhibitory concentration (IC_50_) values of oligomycin, phenformin and AOA were determined using GraphPad Prism 9. FX11 sensitivity was determined empirically, and data were presented by normalizing endpoint confluence to vehicle-treated cells. To calculate cell doubling times, cell confluence area was tracked kinetically on an IncuCyte S3 using phase object confluence for 72 h, and the growth curve was fit to an exponential (Malthusian) growth function using GraphPad Prism 9.

### Amino acid, pyruvate, α-ketobutyrate and nucleoside rescues

Cells were treated in triplicate with either a cocktail containing all NEAAs at 100 µM (Thermo Fisher) or individual amino acids added at a final concentration of 100 µM (Thermo Fisher). Aspartate (20 mM) was prepared directly in DMEM. Pyruvate (Thermo Fisher) was used at 1 mM and α-ketobutyrate at 500 µM. One hour after treatment, oligomycin or vehicle was added. Nucleoside cocktail (Millipore, ES-008-D) was used at 1× concentration. Plates were read 4 d later on the IncuCyte S3 using phase object confluence as a readout. Sensitivity was determined by treating each cell line at the same concentration and normalizing endpoint confluence to vehicle-treated cells.

### Generation of fluorescent cell lines

Lentiviral constructs containing the orange fluorescent marker were generously donated from Essen BioScience, Nuc-Red was a gift from G. Luker and green fluorescent protein (GFP) luciferase was a gift from E. Abel. Cells were seeded at 250,000 cells per well in clear six-well plates (Corning, 3516) and left to attach overnight. The next day, lentiviral constructs were added with polybrene transfection reagent (Sigma). Medium was changed 24 h later. After 48 h, puromycin was added to select transfected cells. New medium with puromycin was refreshed every 48 h. For the GFP luciferase-labeled cells, infected cells were enriched to a <99% GFP^+^ population by fluorescence-activated cell sorting (FACS).

### FACS

Transduced cells were detached with Accutase (Thermo Fisher), pelleted through centrifugation, resuspended in FACS buffer (PBS + 5% bovine serum albumin and 0.5 mM EDTA) and filtered through a 40-µm cell strainer. Cells were sorted on a FACSAria Fusion (BD) using FACSDiva (v8.0.1), gating on live singlets with strong expression of GFP, as shown in the representative gating strategy in Supplemental Fig. 2.

### Coculture assays

Cells were grown on clear 96-well plates (Thermo Fisher, 167425) at 1,000 total cells per well in triplicate and grown for 4 d. Each well was seeded with 500 fluorescently labeled cells and 500 non-labeled cells and immediately placed on an orbital rocker for 20 min to ensure adequate distribution of cells. Cells were left in the incubator to attach overnight, and inhibitors were added the following day. At endpoint, total fluorescent area per well was read using the IncuCyte S3 with IncuCyte Imager Software (2019B). The GCN2iB and ISRIB proliferation assays were performed with Nuc-Red-labeled cells imaged and analyzed on a Cytation 5 (Biotek) using Gen5 Software (v3.11). The PATC53 clonal experiments were performed with GFP-labeled cells imaged and analyzed on a Cytation 5 (Biotek).

### Asparagine synthetase knockdown

ON-TARGETplus siRNA targeting mouse *Asns* was purchased from Dharmacon (L-047839-01-0005) with ON-TARGETplus non-targeting siRNAs as a control (D-001810-10). Cell lines were transfected in six-well plates using Lipofectamine RNAiMAX transfection reagent (Thermo Fisher) per the manufacturer’s instructions. Coculture viability assays were performed on the IncuCyte S3 as described. Parallel transfected and non-transfected cell lines were collected for protein analysis to confirm *Asns* knockdown via western blotting.

### Western blotting

Lysates were quantified by bicinchoninic acid assay (Thermo Fisher Scientific), and equal protein amounts were run on SDS–PAGE gels. Proteins were transferred from SDS–PAGE gels to Immobilon-FL PVDF membranes, blocked and incubated with primary antibodies. After washing, membranes were incubated in secondary antibody, washed and exposed on a Bio-Rad Chemidoc using Bio-Rad Imager Software (v6.1) with West Pico ECL (Thermo Fisher Scientific). Quantitation was done using ImageJ v1.52a.

### Antibodies

The antibodies used in this study are detailed in the Reporting Summary.

### Transwell colony-forming assay

Cells visualized for colonies were grown on clear 24-well plates (Corning, CLS3527) at 200 cells per well for 10 d. Transwell cells were grown on inserts (6.5 mm, 4-µm pore size, polyester) at 1,000 cells per insert (Corning, 3470). Oligomycin (0.25 nM) or vehicle was refreshed every 48 h. Colonies were fixed with 100% methanol, stained with 0.5% crystal violet and rinsed six times with water. Colonies were imaged with a Bio-Rad Chemidoc, and area was quantified using ImageJ.

### Gene set enrichment analysis

DESeq2 analysis was performed on RNA-sequencing data from the clones to identify differentially expressed genes (DEGs) between the two metabolic classes, group 1 (V, E and H) and group 2 (K, M, N and T). Significant DEGs were selected with an adjusted *P* value cutoff of <0.1. Preranked gene set enrichment analysis was then performed on the significant DEG list to identify hallmark gene set enrichment in group 1 and group 2. Negative normalized enrichment scores indicate gene set enrichment in group 1, while positive normalized enrichment scores indicate gene set enrichment in group 2. A false discovery rate *q* value of <0.25 and a nominal *P* value of <0.1 were used to determine significantly enriched hallmark gene sets in both groups.

### Single-cell RNA-sequencing analysis

Human data were obtained from previously published deidentified datasets (GSE155698). PDA human tumor cells (identified by established lineage markers including *KRT18*, *KRT19*, *MUC1*, *TFF1* and *KRT8*) were processed using the Seurat pipeline in R. Dimensionality reduction was performed in Seurat (V3.1.4) via principal-component analysis on the top 3,000 highly variable genes followed by uniform manifold approximation and projection (UMAP) visualization using the top 30 significant components. Clustering was performed using a Louvain algorithm until distinct populations were identified by differentially expressed marker genes (resolution of 1.2), and metabolic gene signatures were visualized using pheatmap in R. Code will be deposited on GitHub after publication.

### Tumor model

KPC7940B cells (2 × 10^6^) were suspended in a 1:1 Matrigel:DMEM mixture and injected subcutaneously in the flanks of syngeneic mice, and 5 × 10^4^ KPC7940B or KPC-MT3 cells were suspended in a 1:1 Matrigel:DMEM mixture and injected orthotopically into C57BL/6J mice (Jackson Laboratories). A total of 1 × 10^6^ clone N cells, 1 × 10^6^ clone V cells or 5 × 10^5^ each of clone N and V cells were injected subcutaneously into the flanks of Nu/Nu mice (Jackson Laboratories). Mice were maintained in specific pathogen-free housing with access to standard diet (Irradiated 5Lod (LabDiet)) and water ad libitum at constant ambient temperature and a 12-h light cycle. Female mice 8 weeks of age were used for tumor implantation experiments and randomized into treatment arms, and all experiments were conducted in accordance with the Office of Laboratory Animal Welfare and approved by the Institutional Animal Care and Use Committees of the University of Michigan and the University of California, Irvine. Maximum tumor burden was limited to 2 cm in any direction, and this metric was not exceeded in this study. Animal numbers were determined in previous work^13^. Mice were excluded from analysis in the case of ulceration or other unrelated complications before the experimental endpoint. Phenformin was administered in drinking water containing 5 mg ml^–1^ sucralose, as previously described, and PEGylated asparaginase (Oncaspar) was injected intraperitoneally at 2 UI per 100 µl of PBS every 72 h.

### Histology

Mice were killed by CO_2_ asphyxiation, and tissue was quickly collected and fixed overnight at room temperature with Z-fix solution (Anatech). Tissues were processed using a Leica ASP300S tissue processor, paraffin embedded and cut into 5-µm sections. Immunohistochemistry was performed on a Discovery Ultra XT autostainer (Ventana Medical Systems) and counterstained with hematoxylin. Hematoxylin and eosin staining were performed per the manufacturer’s instructions. Human PDA tissue was obtained from deidentified individuals through the University of Michigan Central Biorepository.

### Statistics and reproducibility

All experiments were run a minimum of two times with at least three biological replicates, with the exception of the metabolomics studies, which were run once using samples prepared from biological replicates (*n* = 3). Statistics were performed using GraphPad Prism 9 (GraphPad Software). Groups of two were analyzed by two-tailed Student’s *t*-test or two-tailed Mann–Whitney test, and groups greater than two were compared by one-way analysis of variance (ANOVA) with a Tukey post hoc test. All error bars, group numbers and explanation of significant values are presented within the figure legends. The following values are used to denote significance: *P ≤ 0.05, **P ≤ 0.01, ***P ≤ 0.001 and ****P ≤ 0.0001. If multiple significance values were included in a comparison, the least significant comparison was indicated in the figure; all exact *P* values can be found in the Source Data tables. Sample sizes were determined by previous experiments performed in our groups. No data were excluded from the analyses. The experiments were not randomized, and investigators were not blinded to allocation during experiments and outcome assessment. Data distribution was assumed to be normal, but this was not formally tested.

### Reporting summary

Further information on research design is available in the Nature Portfolio Reporting Summary linked to this article.

## Supplementary information

Supplementary Information Supplementary Figs. 1 and 2.

Reporting Summary

Supplementary Table 1 Supplementary Tables 1–4.

## Data Availability

Clonal cell line (mouse PDA clones V, E, H, K, M, N and T) RNA-sequencing data have been deposited in the NCBI’s Gene Expression Omnibus (GEO) database and can be accessed through GEO SuperSeries accession number GSE135436. Human data were reanalyzed from deidentified single-cell RNA-sequencing data available from the NIH GEO database under the accession number GSE155698. Other data that support the findings of this study are available from the corresponding authors upon reasonable request. Source data are provided with this paper.

## Source data

Source Data Fig. 1 Statistical source data.
Source Data Fig. 2 Statistical source data.
Source Data Fig. 3 Statistical source data.
Source Data Fig. 4 Statistical source data.
Source Data Fig. 4 Unprocessed western blots.
Source Data Fig. 5 Statistical source data.
Source Data Fig. 5 Unprocessed western blots.
Source Data Fig. 6 Statistical source data.
Source Data Fig. 7 Statistical source data.
Source Data Extended Data Fig. 1 Unprocessed western blots.
Source Data Extended Data Fig. 1 Statistical source data.
Source Data Extended Data Fig. 2 Statistical source data.
Source Data Extended Data Fig. 4 Statistical source data.
Source Data Extended Data Fig. 5 Statistical source data.
Source Data Extended Data Fig. 5 Unprocessed western blots.
Source Data Extended Data Fig. 6 Unprocessed western blots.
Source Data Extended Data Fig. 8 Statistical source data.
Source Data Extended Data Fig. 8 Unprocessed western blots.
Source Data Extended Data Fig. 10 Statistical source data.

## Code availability

Code for the single-cell analyses has been deposited at GitHub at https://github.com/halbrook/HalbrookLab/blob/main/Clonal%20Heterogeneity%20NatCan2022.R.
