# Supplementary material for: Differential integrated stress response and asparagine production drive symbiosis and therapy resistance of pancreatic adenocarcinoma cells
Source: Nat Cancer. 2022 Nov 21;3(11):1386–403. doi: 10.1038/s43018-022-00463-1 (PMC9701142; doi:10.1038/s43018-022-00463-1)
Supplement: Supplementary file 1 — Supplementary Figs. 1 and 2. [file 43018_2022_463_MOESM1_ESM.pdf]

nature cancer

Article

https://doi.org/10.1038/s43018-022-00463-1

Differential integrated stress response and asparagine production drive symbiosis and therapy resistance of pancreatic adenocarcinoma cells

In the format provided by the authors and unedited

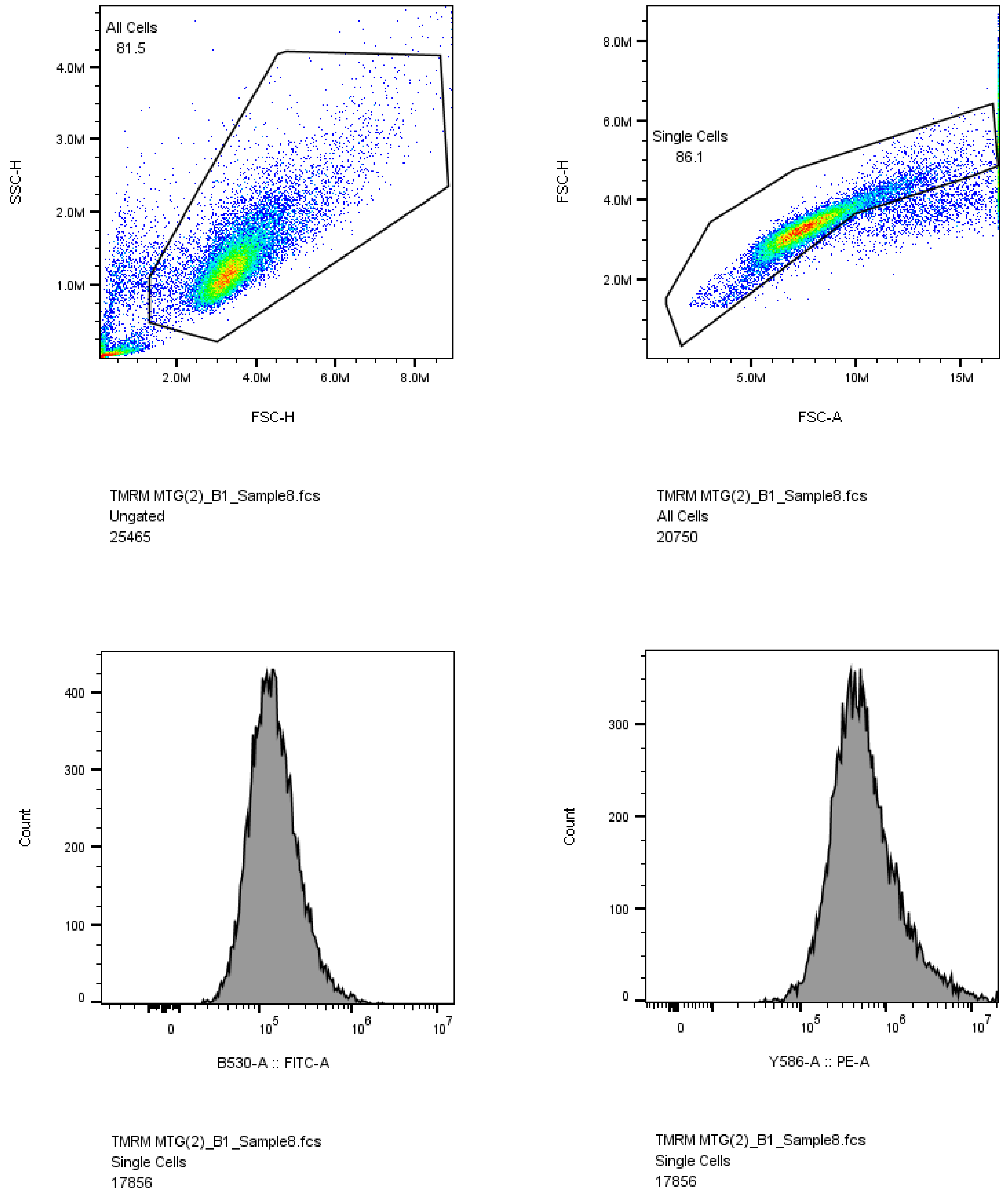

Supplemental Figure 1: Mitochondrial staining gating strategy. Cells were gated on singlets and then fluorescence recorded in the FITC and PE A channels.

BD FACSDiva 8.0.1

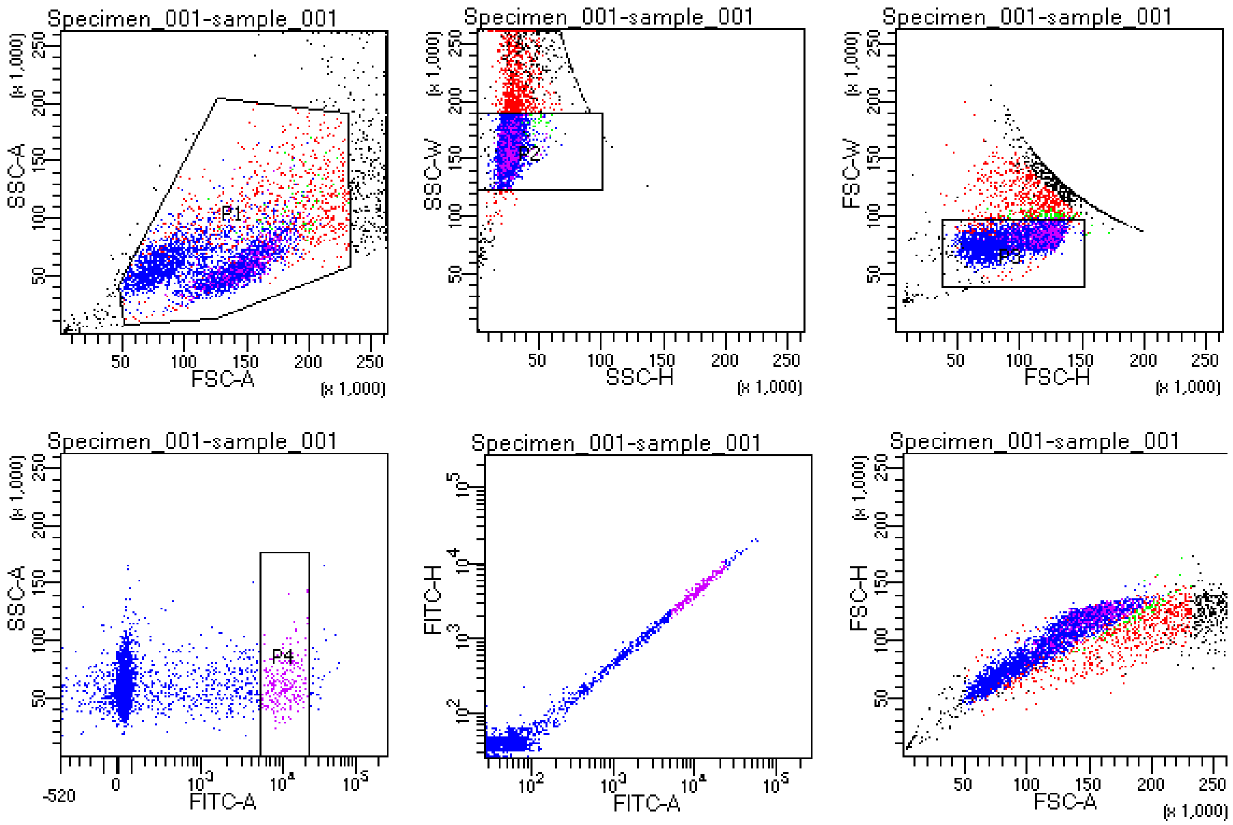

Tube: sample_001

Population	#Events	%Parent	%Total
All Events	5,355	####	100.0
P1	4,041	75.5	75.5
P2	3,179	78.7	59.4
P3	3,114	98.0	58.2
P4	247	7.9	4.6
P5	0	0.0	0.0

Supplemental Figure 2: FACS sorting strategy. Cells were first gated on singlets, then gated on GFP fluorescence through the FITC channel. The top 2% brightests were excluded, and the next 8% brightest sorted.
