# Supplementary material for: Differential integrated stress response and asparagine production drive symbiosis and therapy resistance of pancreatic adenocarcinoma cells
Source: Nat Cancer. 2022 Nov 21;3(11):1386–403. doi: 10.1038/s43018-022-00463-1 (PMC9701142; doi:10.1038/s43018-022-00463-1)
Supplement: Supplementary file 2 — Reporting Summary [file 43018_2022_463_MOESM2_ESM.pdf]

nature portfolio

Corresponding author(s): Lyssiotis, CA; Halbrook, CJ

Last updated by author(s): Oct 3, 2022

Reporting Summary

Nature Portfolio wishes to improve the reproducibility of the work that we publish. This form provides structure for consistency and transparency in reporting. For further information on Nature Portfolio policies, see our Editorial Policies and the Editorial Policy Checklist.

Statistics

For all statistical analyses, confirm that the following items are present in the figure legend, table legend, main text, or Methods section.

n/a	Confirmed	
☐	☒	The exact sample size (*n*) for each experimental group/condition, given as a discrete number and unit of measurement
☐	☒	A statement on whether measurements were taken from distinct samples or whether the same sample was measured repeatedly
☐	☒	The statistical test(s) used AND whether they are one- or two-sided<br>*Only common tests should be described solely by name; describe more complex techniques in the Methods section.*
☐	☒	A description of all covariates tested
☐	☒	A description of any assumptions or corrections, such as tests of normality and adjustment for multiple comparisons
☐	☒	A full description of the statistical parameters including central tendency (e.g. means) or other basic estimates (e.g. regression coefficient) AND variation (e.g. standard deviation) or associated estimates of uncertainty (e.g. confidence intervals)
☐	☒	For null hypothesis testing, the test statistic (e.g. *F*, *t*, *r*) with confidence intervals, effect sizes, degrees of freedom and *P* value noted<br>*Give P values as exact values whenever suitable.*
☒	☐	For Bayesian analysis, information on the choice of priors and Markov chain Monte Carlo settings
☒	☐	For hierarchical and complex designs, identification of the appropriate level for tests and full reporting of outcomes
☐	☒	Estimates of effect sizes (e.g. Cohen's *d*, Pearson's *r*), indicating how they were calculated

Our web collection on statistics for biologists contains articles on many of the points above.

Software and code

Policy information about availability of computer code

Data collection	Agilent Mass Spectrometry software (vB0700), BioRad Imager Software (v6.1), Gen5 Cytation Software (v3.11), FACSDiva (v8.0.1), NovoExpress flow cytometry software (version 1.6.0), and IncuCyte Imager Software (2019B) were all used as described in the methods.
Data analysis	Image J (v1.52a), FloJo (v10.8), Morpheus Matrix Visualization and analysis tool, and GraphPad Prism 9 were used as described in the methods.

For manuscripts utilizing custom algorithms or software that are central to the research but not yet described in published literature, software must be made available to editors and reviewers. We strongly encourage code deposition in a community repository (e.g. GitHub). See the Nature Portfolio guidelines for submitting code & software for further information.

Data

Policy information about availability of data

All manuscripts must include a data availability statement. This statement should provide the following information, where applicable:

- Accession codes, unique identifiers, or web links for publicly available datasets
- A description of any restrictions on data availability
- For clinical datasets or third party data, please ensure that the statement adheres to our policy

Clonal cell line (murine PDA clones V, E, H, K, M, N, T) RNA sequencing data has been deposited in the NCBI's Gene Expression Omnibus database and can be accessed through GEO SuperSeries accession number GSE135436. Human patient data was re-analyzed from deidentified scRNA-seq data available from the NIH

Gene Expression Omnibus database under the accession number GSE155698. Other data that support the findings of this study are available from the corresponding authors upon reasonable request.
Code for the single-cell analyses has been deposited at Github at https://github.com/halbrook/HalbrookLab/blob/main/Clonal%20Heterogeneity%20NatCan2022.R.

Human research participants

Policy information about studies involving human research participants and Sex and Gender in Research.

Reporting on sex and gender	Sex and gender were not provided or used in the selection of slides for histological analysis.
Population characteristics	University of Michigan patients with confirmed Pancreatic Ductal Adenocarcinoma eligible for surgical resection.
Recruitment	Deidentified samples from the tissue repository supported by IRB (HUM00025339) were used for histological analysis.
Ethics oversight	University of Michigan Institutional Review Board (HUM00025339).

Note that full information on the approval of the study protocol must also be provided in the manuscript.

Field-specific reporting

Please select the one below that is the best fit for your research. If you are not sure, read the appropriate sections before making your selection.

☒ Life sciences ☐ Behavioural & social sciences ☐ Ecological, evolutionary & environmental sciences

For a reference copy of the document with all sections, see nature.com/documents/nr-reporting-summary-flat.pdf

Life sciences study design

All studies must disclose on these points even when the disclosure is negative.

Sample size	In vivo study numbers were determined by a power analysis assuming a power of 90% (0.90) and a 5% significance level (alpha = 0.05). Our Cohen's coefficient was d=2 and was empirically derived using values from previous iterations of these experiments (see Halbrook et al., Cell Metabolism 2019).
Data exclusions	No data was excluded from analyses in this manuscript.
Replication	All experiments were successfully reproduced a minimum of three times with at least 3 biological replicates, with the exception of the metabolomics studies which were run once using samples prepared from biological replicates (n=3).
Randomization	Tumor bearing mice were randomized onto treatment arms.
Blinding	Blinding was not possible as the treatments were administered by the same party responsible for data collection.

Reporting for specific materials, systems and methods

We require information from authors about some types of materials, experimental systems and methods used in many studies. Here, indicate whether each material, system or method listed is relevant to your study. If you are not sure if a list item applies to your research, read the appropriate section before selecting a response.

Materials & experimental systems

n/a	Involved in the study	
☐	☒	Antibodies
☐	☒	Eukaryotic cell lines
☒	☐	Palaeontology and archaeology
☐	☒	Animals and other organisms
☒	☐	Clinical data
☒	☐	Dual use research of concern

Methods

n/a	Involved in the study	
☒	☐	ChIP-seq
☐	☒	Flow cytometry
☒	☐	MRI-based neuroimaging

Antibodies

Antibodies used	Asparagine synthetase Antibody (G-10) sc-365809 Santa Cruz 1:1000 (Western Blot)

Antibodies used

Asparagine synthetase antibody 14681-1-AP Proteintech 1:250 (Immunohistochemistry)
ATF-4 (D4B8) Rabbit mAb #11815 Cell Signaling 1:1000
ATF-6 antibody #75478 Novus 1:1000
SHMT2 Antibody #12762 Cell Signaling 1:1000
Phospho-GCN2 (Thr899) (E1V9M) #94668 Cell Signaling 1:500
IRE1a Antibody (14C10) #3294 Cell Signaling 1:1000
Anti-IRE1 (phospho S724) antibody #ab48187 Abcam 1:1000
HRI Antibody #MBS2538144 MyBioSource 1:1000
Phospho-PERK-T982 #AP0886 AbClonal 1:1000
PERK antibody (C33E10) #3192 Cell Signaling 1:1000
Anti-Phospho-PKR (Thr446) #37704 Invitrogen 1:1000
PKR Antibody #3072 Cell Signaling 1:1000
Phospho-p44/42 MAPK (Erk1/2) (Thr202/Tyr204) (E10) Mouse mAb #9106 Cell Signaling 1:1000
p44/42 MAPK (Erk1/2) (137F5) Rabbit mAb #4695 Cell Signaling 1:1000
c-Myc (D84C12) #5605 Cell Signaling 1:1000
Slug (C19G7) Rabbit mAb #9585 Cell Signaling 1:1000
HIF-1α (D1S7W) XP® Rabbit mAb #36169 Cell Signaling 1:1000
Cleaved Caspase-3 (Asp175) (5A1E) Rabbit mAb #9664 Cell Signaling 1:1000
Vinculin (E1E9V) XP #13901 Cell Signaling 1:5000
Anti-rabbit IgG, HRP-linked Antibody #7074 Cell Signaling 1:5000
Anti-mouse IgG, HRP-linked Antibody #7076 Cell Signaling 1:5000

Validation

All antibodies were validated by their commercial sources as indicated below:

Asparagine synthetase Antibody (G-10) sc-365809 Santa Cruz, Asparagine synthetase Antibody (G-10) is a high quality monoclonal Asparagine synthetase antibody suitable for the detection of the Asparagine synthetase protein of mouse, rat and human origin. Antibody is suitable for western blot, immunohistochemistry, immunoprecipitation, and immunofluorescence.
Asparagine synthetase antibody 14681-1-AP Proteintech is validated in human and predicted to react with mouse and rat. It is suitable for Western Blot, immunoprecipitation, and immunofluorescence.
ATF-4 (D4B8) Rabbit mAb #11815 Cell Signaling recognizes endogenous levels of total ATF-4 protein. Species Reactivity: Human, Mouse, Rat. Validated applications: Western Blotting, Immunoprecipitation, Immunofluorescence (Immunocytochemistry), Chromatin IP, Chromatin IP-seq, CUT&RUN
ATF-6 antibody #75478 Novus is suitable for detection of Human and Mouse ATF6. Suitable applications include Western Blot, Simple Western, ICC/IF.
SHMT2 Antibody #12762 Cell Signaling antibody recognizes endogenous levels of total SHMT2 protein. Species Reactivity include Human, Mouse, Rat, Monkey. It is suitable for western blotting.
Phospho-GCN2 (Thr899) (E1V9M) #94668 Cell Signaling rabbit mAb recognizes endogenous levels of GCN2 protein only when phosphorylated at Thr899. Species Reactivity: Human Species predicted to react based on 100% sequence homology: Mouse, Rat. Suitable applications Western Blotting, Immunofluorescence (Immunocytochemistry), Flow Cytometry (Fixed/Permeabilized).
IRE1a Antibody (14C10) #3294 Cell Signaling rabbit mAb detects endogenous levels of total IRE1α protein. Species Reactivity: Human, Mouse, Rat. Suitable applications western blot, immunoprecipitation.
Anti-IRE1 (phospho S724) antibody #ab48187 Abcam Suitable for: WB, ELISA, IHC-P
Reacts with: Mouse, Human, Recombinant fragment.
HRI Antibody #MBS2538144 MyBioSource is a Rabbit polyclonal antibody. Species Reactivity includes Human, Mouse, Monkey. Suitable for Western Blot (WB), Immunohistochemistry-paraffin (IHC-p), and ELISA (EIA).
Phospho-PERK-T982 #AP0886 AbClonal is a rabbit polyclonal antibody. Cross-Reactivity Human, Mouse, Rat. Suitable application: Western Blot.
PERK antibody (C33E10) #3192 Cell Signaling is a rabbit monoclonal antibody reactive to Human, Mouse, Rat, and Monkey. Suitable application is western blot.
Anti-Phospho-PKR (Thr446) #37704 Invitrogen is a rabbit polyclonal antibody suitable for western blots, reactive to human.
PKR Antibody #3072 Cell Signaling is a rabbit polyclonal antibody rabbit suitable for immunoprecipitation and western blots, reactive to human.
Phospho-p44/42 MAPK (Erk1/2) (Thr202/Tyr204) (E10) Mouse mAb #9106 Cell Signaling is reactive to Human, Mouse, Rat, Hamster, Monkey, Mink, Zebrafish, Bovine, Pig. It is suitable for flow cytometry and western blotting.
p44/42 MAPK (Erk1/2) (137F5) Rabbit mAb #4695 Cell Signaling is reactive to Human, Mouse, Rat, Hamster, Monkey, Mink, Zebrafish, Bovine, Pig, D. melanogaster, Dog, and C. elegans. Suitable applications include western blotting, immunoprecipitation, immunohistochemistry, immunfluorescence, and flow cytometry.
c-Myc (D84C12) #5605 Cell Signaling is reactive to human, mouse, and rat. Appropriate applications include western blotting and immunofluorescence.
Slug (C19G7) Rabbit mAb #9585 Cell Signaling is reactive to human and mouse. Appropriate applications include western blotting and immunofluorescence, and flow cytometry.
HIF-1α (D1S7W) XP® Rabbit mAb #36169 Cell Signaling is reactive to human, mouse, and monkey. Suitable applications include western blotting, immunoprecipitation, immunohistochemistry, immunfluorescence, flow cytometry, chromatin IP, and CUT and Run.
Cleaved Caspase-3 (Asp175) (5A1E) Rabbit mAb #9664 Cell Signaling is reactive to human, mouse, rat, and monkey. Appropriate applications include western blotting and immunofluorescence, and flow cytometry.
Vinculin (E1E9V) XP #13901 Cell Signaling is reactive to human, mouse, rat, and monkey. It is suitable for western blotting, immunohistochemistry, and flow cytometry.
Anti-rabbit IgG, HRP-linked Antibody #7074 Cell Signaling is designed for use with rabbit polyclonal and monoclonal antibodies, this affinity purified goat anti-rabbit IgG (heavy and light chain) antibody is conjugated to horseradish peroxidase(HRP) for chemiluminescent detection. This product is thoroughly validated with CST primary antibodies.
Anti-mouse IgG, HRP-linked Antibody #7076 Cell Signaling is an affinity purified horse anti-mouse IgG (heavy and light chain) antibody is conjugated to horseradish peroxidase(HRP) for chemiluminescent detection. This product is thoroughly validated with CST primary antibodies.

Eukaryotic cell lines

Policy information about cell lines and Sex and Gender in Research

Cell line source(s)	Clonal cell lines E, H, K, M, N, T, V were derived from murine KrasG12D; PDX-1Cre, Trp53R172H (KPC) tumors and previously reported (doi:http://dx.doi.org/10.2139/ssrn.3486019.). Clonal lines 6419c5, 6694c2, 2838c3, and 6499c4 were derived from KPC tumors and previously described (doi:10.1016/j.immuni.2018.06.006). PATC53 clones were obtained commercially through ATCC.
Authentication	Cell lines were not authenticated commercially.
Mycoplasma contamination	All cell lines were routinely tested for mycoplasma.
Commonly misidentified lines (See ICLAC register)	No cell lines on this list were used to generate any data presented in this manuscript.

Animals and other research organisms

Policy information about studies involving animals; ARRIVE guidelines recommended for reporting animal research, and Sex and Gender in Research

Laboratory animals	C57BL/6J, and Nu/Nu mice were obtained from Jackson Laboratories and used at 8 weeks of age. Mice were maintained in specific pathogen-free housing with access to standard diet (Irradiated 5Lod) and water ad libitum at constant ambient temperature and a 12-hour light cycle.
Wild animals	This study did not involve wild animals.
Reporting on sex	Female animals were used for tumor studies, as outlined in the methods, and sex was not considered in the study design.
Field-collected samples	This study did not involve field-collected samples.
Ethics oversight	The animal experiments in this study were performed in compliance with the Institutional Animal Care and Use Committees of the University of Michigan (PRO00008877, Lyssiotis) and the University of California, Irvine (AUP-20-102, Halbrook).

Note that full information on the approval of the study protocol must also be provided in the manuscript.

Flow Cytometry

Plots

Confirm that:

☒ The axis labels state the marker and fluorochrome used (e.g. CD4-FITC).

☒ The axis scales are clearly visible. Include numbers along axes only for bottom left plot of group (a 'group' is an analysis of identical markers).

☒ All plots are contour plots with outliers or pseudocolor plots.

☒ A numerical value for number of cells or percentage (with statistics) is provided.

Methodology

Sample preparation	Transduced cells were detached with Accutase (Thermo Fisher), pelleted through centrifugation, resuspended in FACS buffer (PBS+5%BSA, 0.5mM EDTA) then filtered through a 40µM cell strainer.
Instrument	A BD FACSAria Fusion machine was utilized for FACS, an ACEA NovoCyte Quanteon for mitochondrial characterization.
Software	FACSDiva (v8.0.1) software was used for the FACSAria, NovoExpress flow cytometry software (version 1.6.0) for the NovoCyte.
Cell population abundance	For FACS, GFP positive cells ~10% of live cells) were enriched from bulk transduced population, and verified to be a 99% GFP+ population post-sort. Singlet cells were used for mitochondrial characterization.
Gating strategy	For FACS, singlet cells were identified by forward and side scatter, than GFP expression used to define the sorting gates to isolate cells strongly expressing GFP, excluding the top 5% of brightest cells to achieve better uniformity of expression. For mitochondrial characterization, singlet cells were analyzed.

☒ Tick this box to confirm that a figure exemplifying the gating strategy is provided in the Supplementary Information.
