# Supplementary material for: Differential integrated stress response and asparagine production drive symbiosis and therapy resistance of pancreatic adenocarcinoma cells
Source: Nat Cancer. 2022 Nov 21;3(11):1386–403. doi: 10.1038/s43018-022-00463-1 (PMC9701142; doi:10.1038/s43018-022-00463-1)
Supplement: Source Data Fig. 4 — Unprocessed western blots. [file 43018_2022_463_MOESM8_ESM.pdf]

Figure 4A

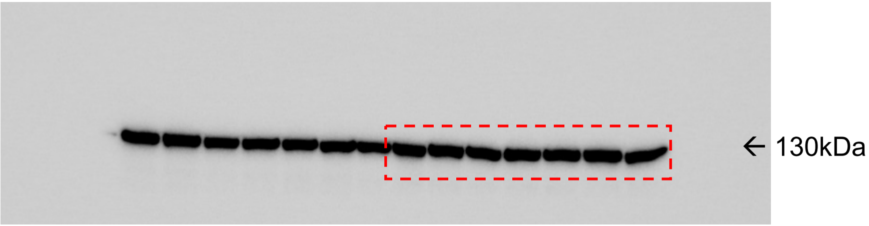

Anti-Vinculin

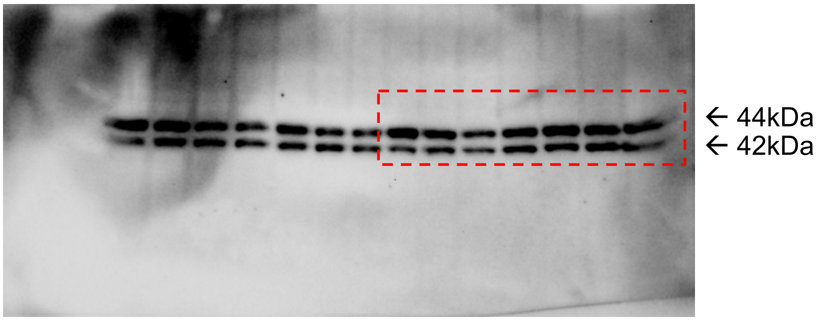

Anti-MAPK (ERK)

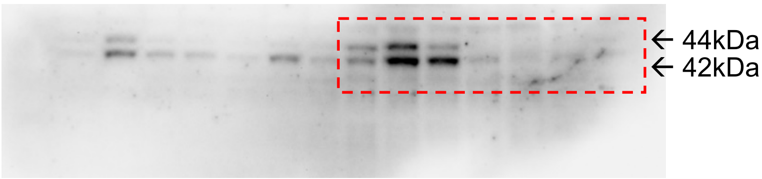

Anti-phospho-MAPK (pERK)

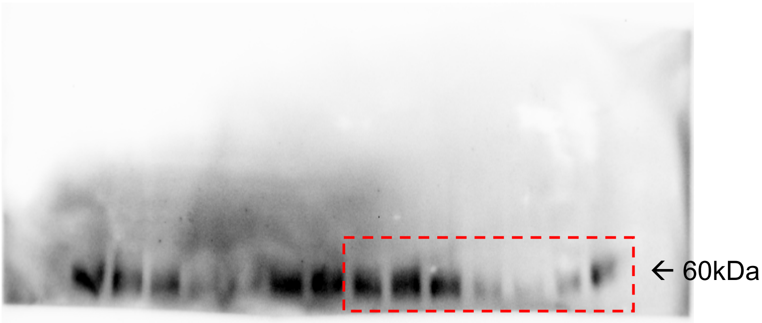

Anti-c-Myc

Figure 4C

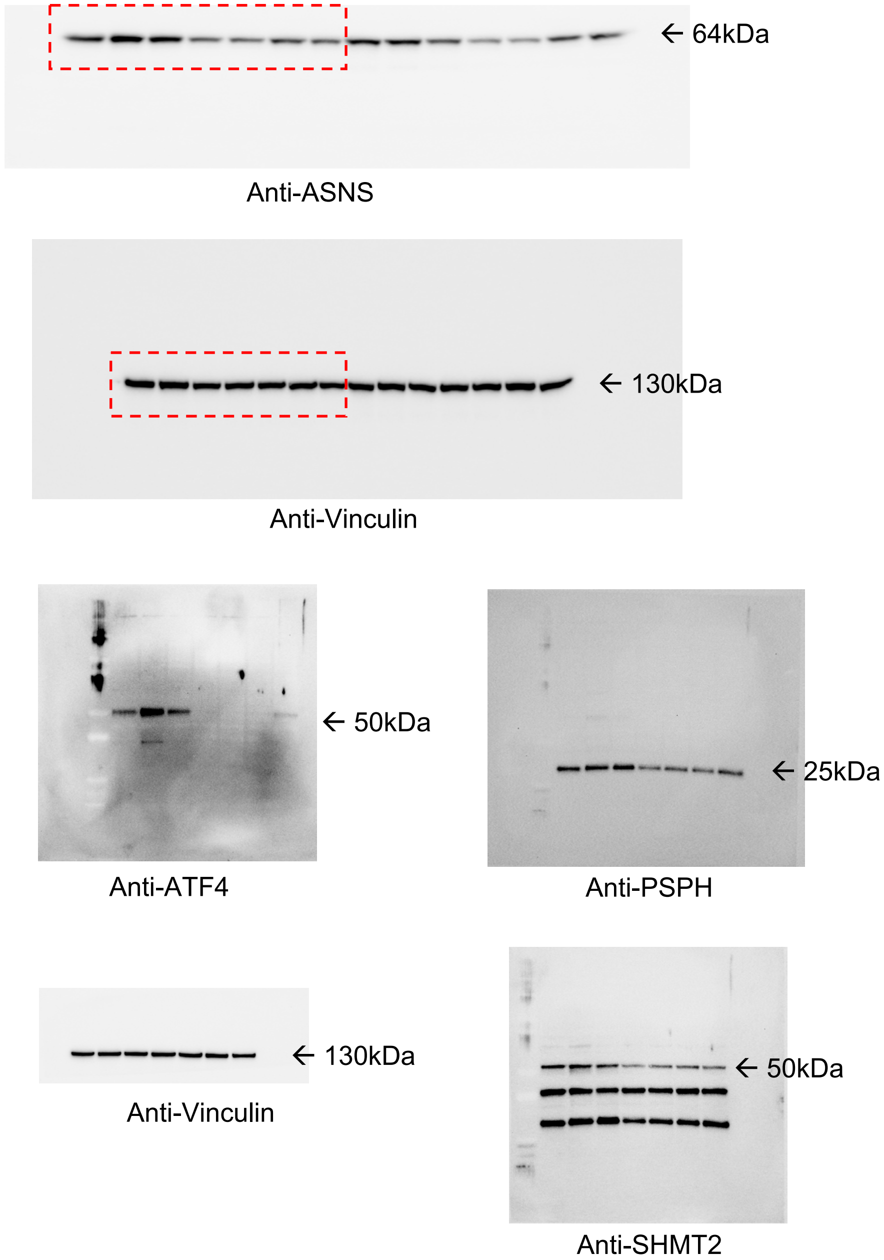

64kDa
Anti-ASNS
130kDa
Anti-Vinculin
50kDa
Anti-ATF4
25kDa
Anti-PSPH
130kDa
Anti-Vinculin
50kDa
Anti-SHMT2

Figure 4E.

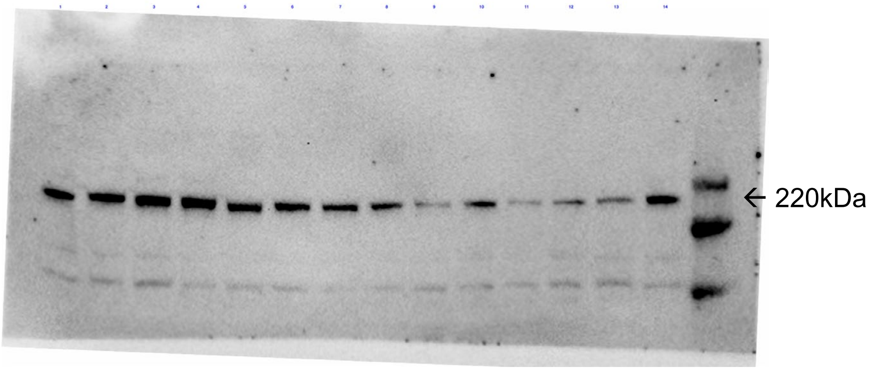

Anti-pGCN2

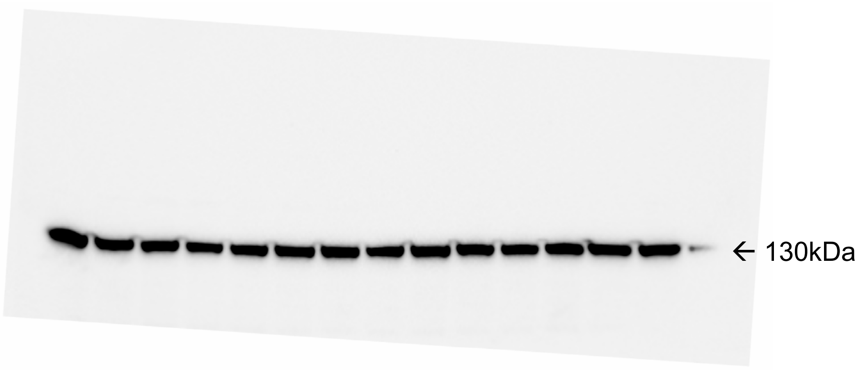

Anti- Vinculin
