# Supplementary figures and images for: Differential integrated stress response and asparagine production drive symbiosis and therapy resistance of pancreatic adenocarcinoma cells
Source: Nat Cancer. 2022 Nov 21;3(11):1386–403. doi: 10.1038/s43018-022-00463-1 (PMC9701142; doi:10.1038/s43018-022-00463-1)

Figure 5E

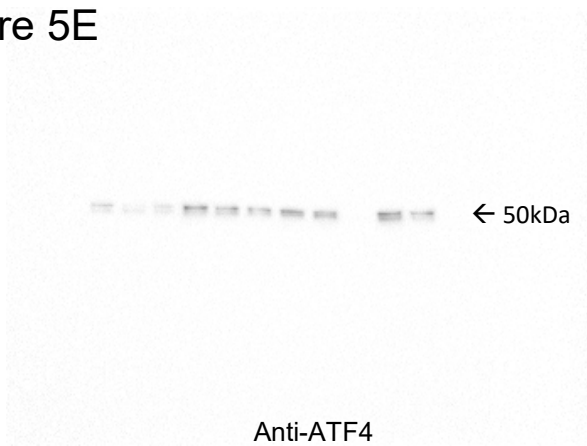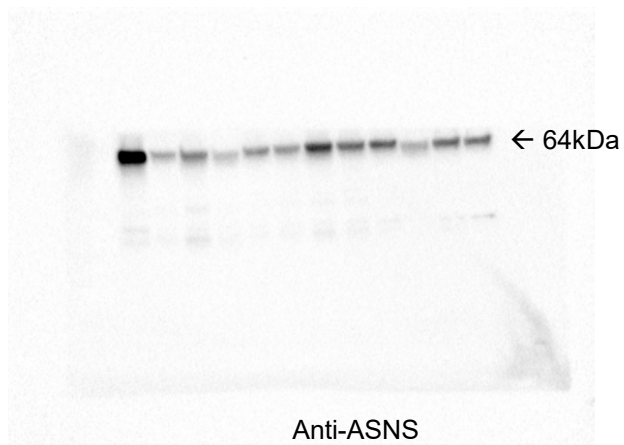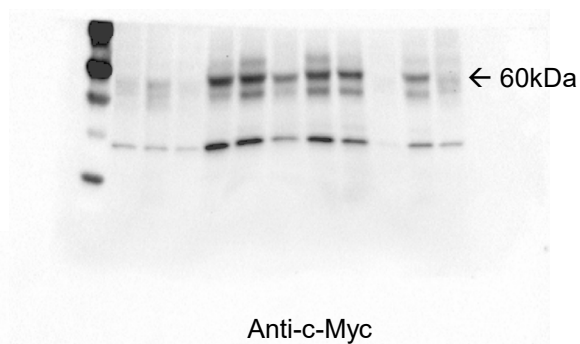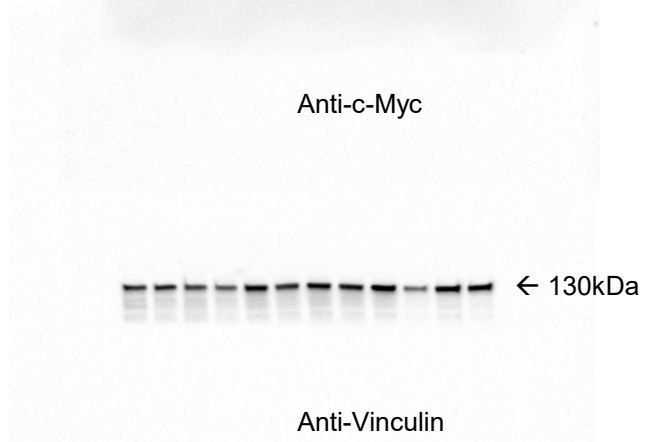

Supplement: Source Data Fig. 5 — Unprocessed western blots. [file 43018_2022_463_MOESM10_ESM.pdf]

Ext. Data Fig. 1a

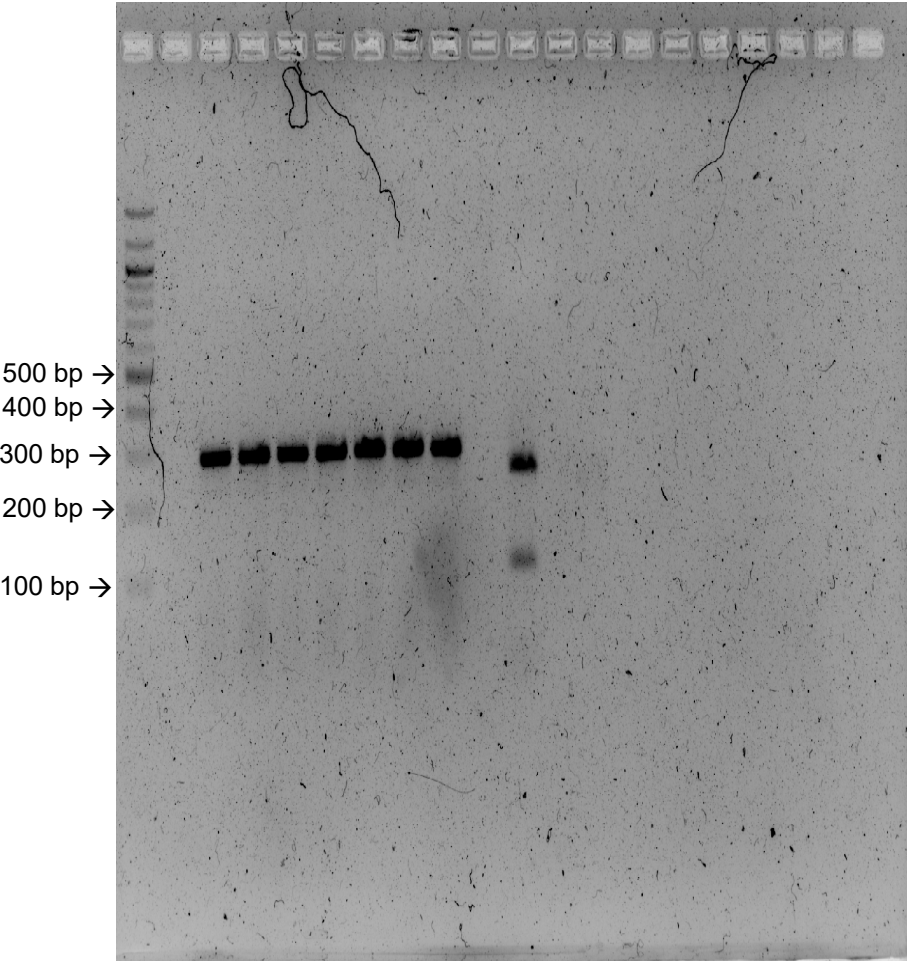

Supplement: Source Data Extended Data Fig. 1 — Statistical source data. [file 43018_2022_463_MOESM14_ESM.pdf]

## Ex. Data Figure 5g

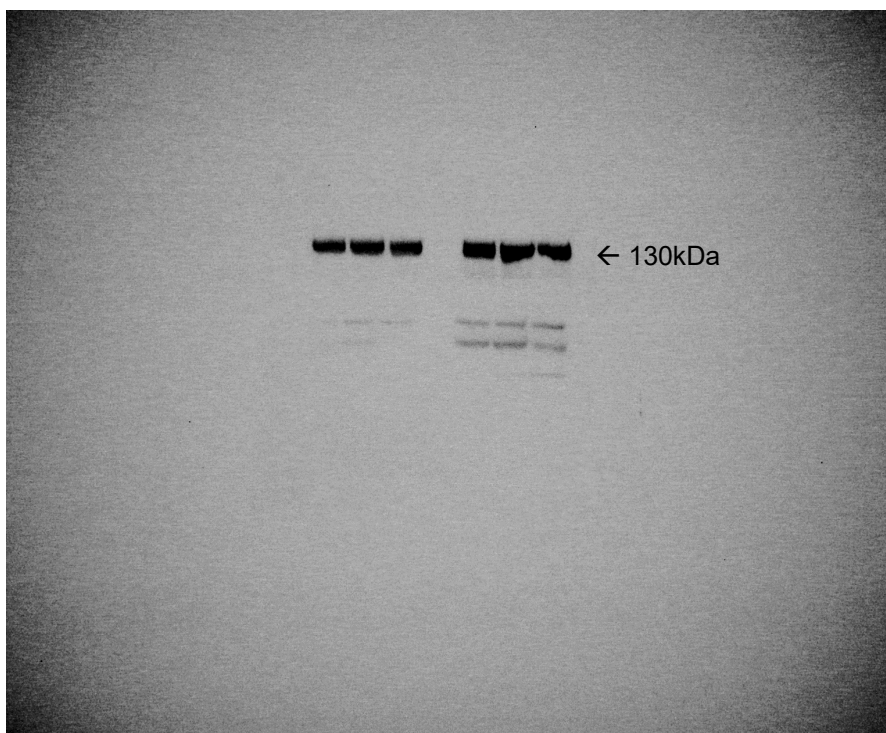

Anti-Vinculin

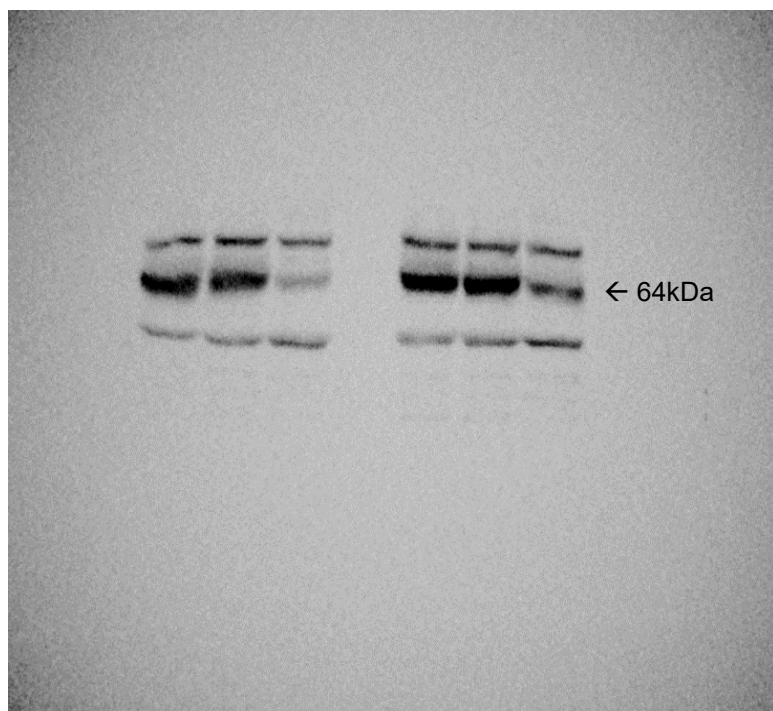

Anti-ASNS

Supplement: Source Data Extended Data Fig. 5 — Unprocessed western blots. [file 43018_2022_463_MOESM18_ESM.pdf]

Extended Data Fig. 8A

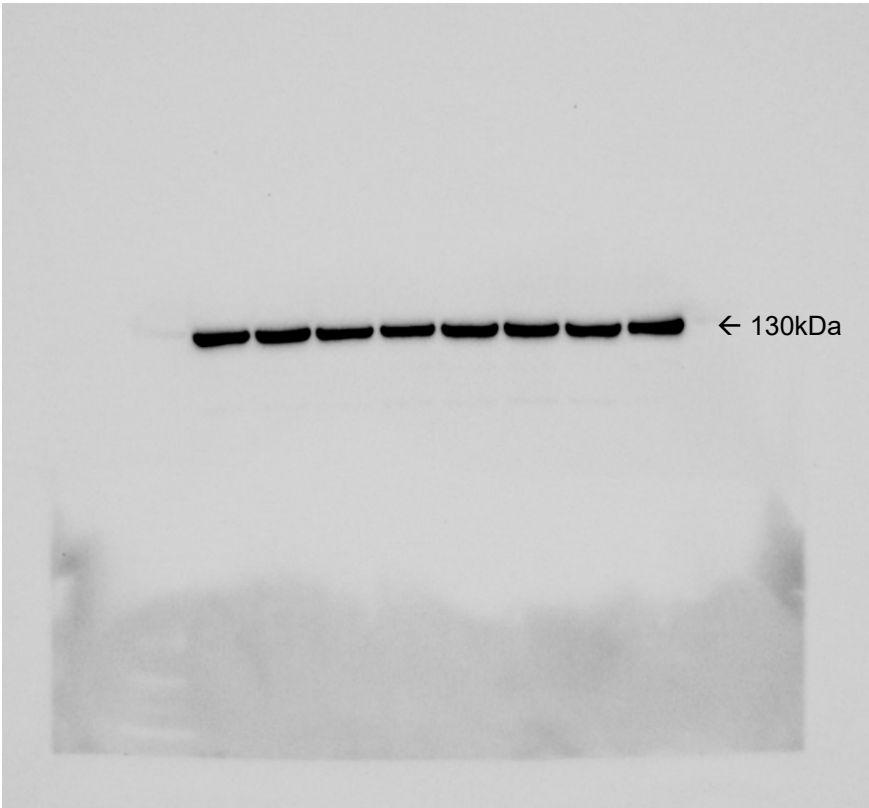

Anti-Vinculin

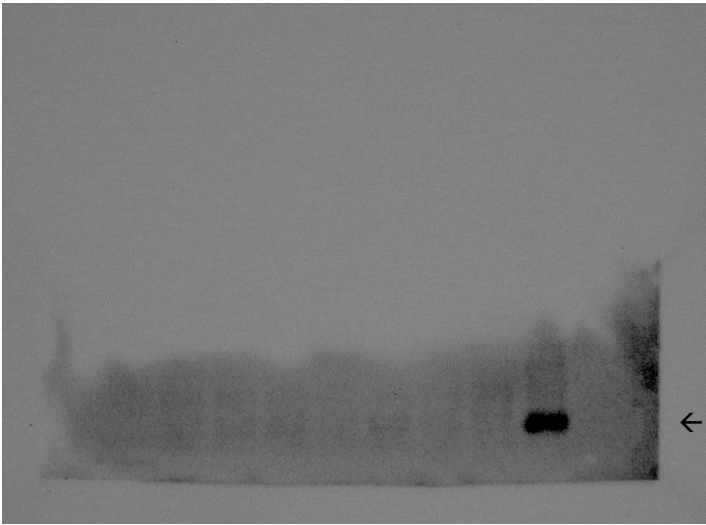

Cleaved Caspase-3

Supplement: Source Data Extended Data Fig. 8 — Unprocessed western blots. [file 43018_2022_463_MOESM21_ESM.pdf]
