# Supplementary material for: Differential integrated stress response and asparagine production drive symbiosis and therapy resistance of pancreatic adenocarcinoma cells
Source: Nat Cancer. 2022 Nov 21;3(11):1386–403. doi: 10.1038/s43018-022-00463-1 (PMC9701142; doi:10.1038/s43018-022-00463-1)
Supplement: Source Data Extended Data Fig. 6 — Unprocessed western blots. [file 43018_2022_463_MOESM19_ESM.pdf]

Ex. Data Figure 6b

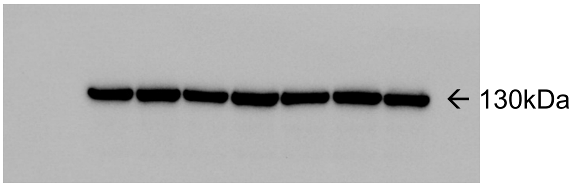

Anti-Vinculin

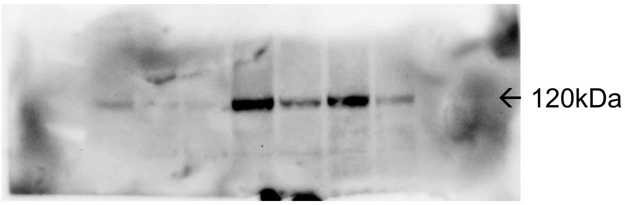

Anti-HIF1a

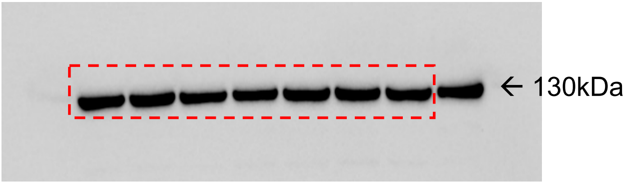

Anti-Vinculin

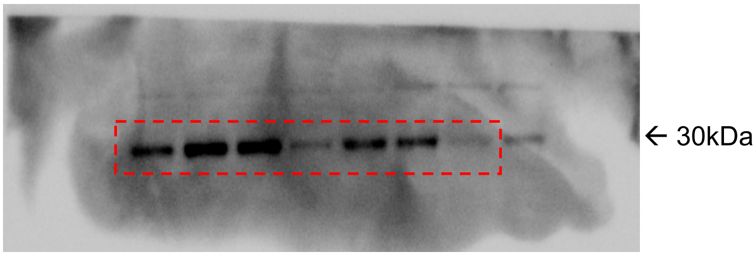

Anti-Slug

Ex. Data Figure 6c

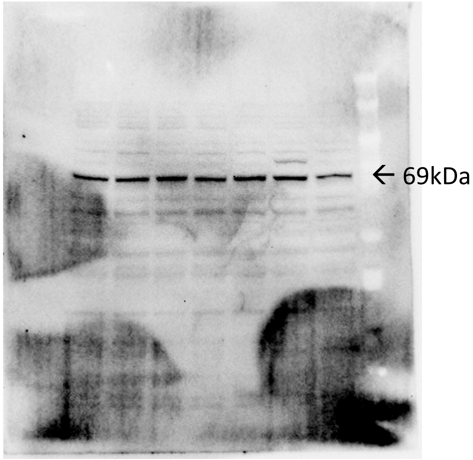

Anti p-PKR

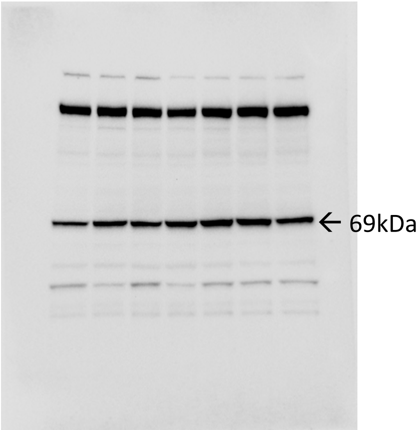

Anti PKR

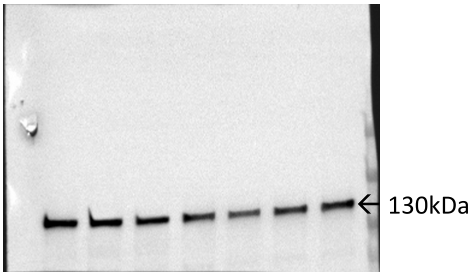

Vinculin

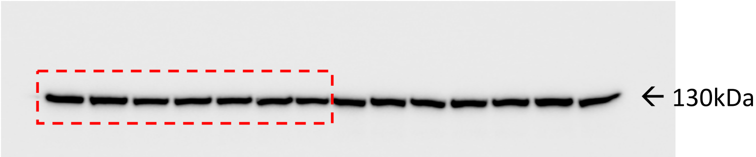

Anti-Vinculin

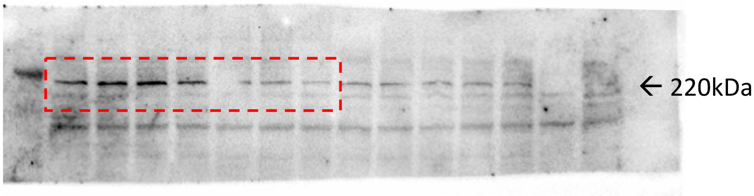

Anti-pGCN2

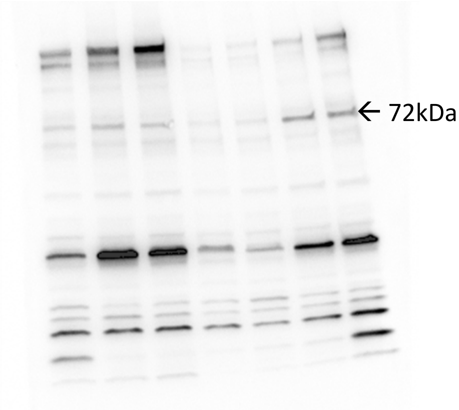

Anti HRI

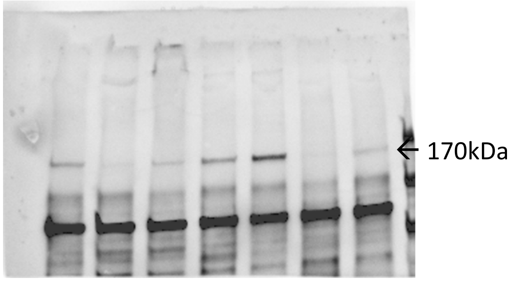

p-PERK

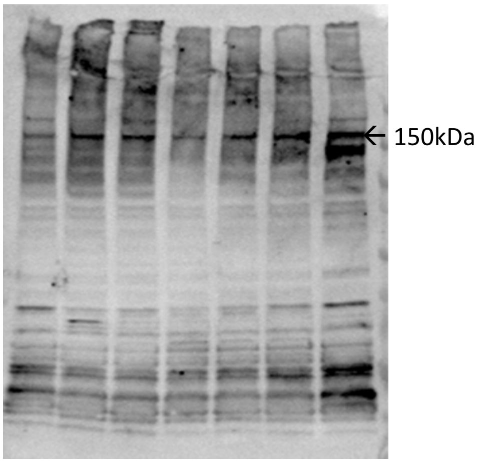

PERK

Ex. Data Figure 6d

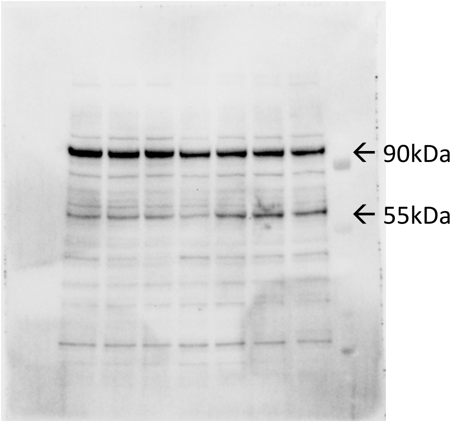

Anti-ATF6

Full-length at 90kDa
Cleaved product at 55kDa

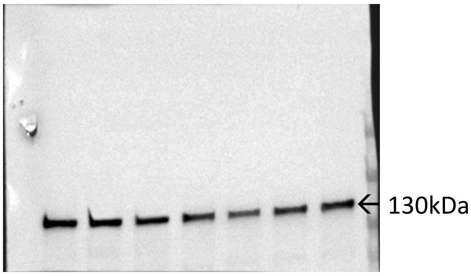

Anti-Vinculin

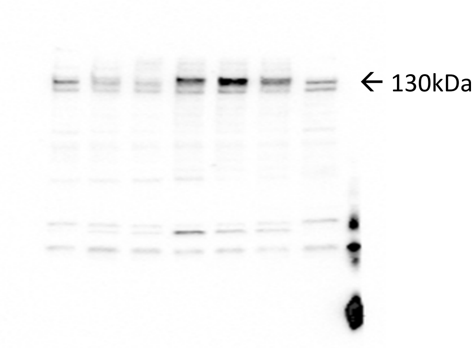

Anti-pIRE1α (S724)

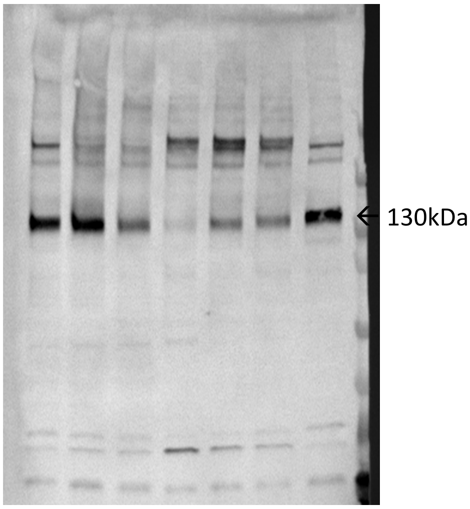

Anti-IRE1α

Ex. Data Figure 6e

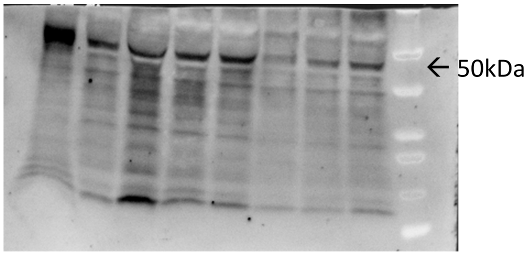

← 50kDa

Anti-ATF4

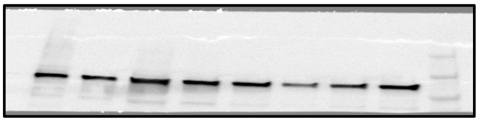

← 130kDa

Anti-Vinculin
